# Advances in Membrane, Dialyzer Design, and Related Monitoring Technologies for Hemodiafiltration: Translating Bench-Side Innovations to Bedside Applications

**DOI:** 10.3390/jcm15051921

**Published:** 2026-03-03

**Authors:** Alfred Gagel, Gerhard Wiesen, Stefano Stuard, Bernard Canaud

**Affiliations:** 1 Global Research and Development, Fresenius Medical Care Deutschland GmbH, 61352 Bad Homburg, Germany; 2 Global Medical Office, Fresenius Medical Care, 26020 Palazzo Pignano, Italy; stefano.stuard@freseniusmedicalcare.com; 3 School of Medicine & AIDER-Santé, Montpellier University, Foundation Ch Mion, 34060 Montpellier, France

**Keywords:** hemodiafiltration, dialysis membrane, convective volume, synthetic polymers, uremic toxin clearance, ultrafiltration, albumin loss, kidney replacement therapy

## Abstract

**Background**: Online hemodiafiltration (HDF) represents the most advanced form of kidney replacement therapy, combining diffusive and convective transport to enhance the removal of uremic toxins across a wide molecular spectrum. Achieving high convective volumes is a key determinant of treatment efficacy and has been associated with improved survival. Beyond small solutes, HDF targets middle molecules and protein-bound uremic toxins (PBUTs), including β_2_-microglobulin, inflammatory cytokines, and other large uremic compounds implicated in cardiovascular and systemic complications. **Aims**: This narrative review examines advances in dialysis membrane materials, dialyzer design, and monitoring technologies that optimize mass transfer in HDF. It focuses on the interplay between membrane permeability, hemocompatibility, and convective dose delivery, and discusses how these engineering developments translate into clinical performance. **Key mechanisms**: Recent progress in synthetic polymer membranes, particularly polysulfone- and polyethersulfone-based systems, and hollow-fiber manufacturing has enabled improved control of pore size distribution, hydraulic permeability, and sieving characteristics. These developments enhance the clearance of middle molecules and selected PBUTs while preserving essential proteins such as albumin. Mechanistic insights into internal filtration, protein polarization, and Donnan effects highlight the complex transport processes occurring within the dialyzer and their interaction with automated HDF systems. Expanded hemodialysis and high-volume HDF approaches further increase the removal of larger solutes but require careful management to limit albumin loss and maintain hemocompatibility. **Clinical implications**: Optimized membrane design, combined with advanced HDF machine algorithms, allows delivery of high convective volumes under safe and stable conditions, improving removal of β_2_-microglobulin, cytokines, and other clinically relevant toxins associated with inflammation and cardiovascular risk. However, treatment must remain individualized, considering electrolyte balance, albumin preservation, and patient-specific factors such as inflammation and nutritional status. Mechanistic modeling supports understanding of transport phenomena but must be interpreted cautiously when translated into clinical practice. **Conclusions**: Advances in membrane science, dialyzer engineering, and monitoring technologies have strengthened the role of HDF as a precision-based renal replacement therapy. Continued innovation aimed at optimizing middle-molecule and PBUT clearance while preserving albumin and treatment stability is essential to improve patient outcomes and support the broader implementation of HDF as a mainstream dialysis modality.

## 1. Introduction

Online hemodiafiltration (HDF) represents the most advanced form of kidney replacement therapy, integrating both diffusive and convective solute transport to effectively remove uremic toxins, especially middle- and large-molecular-weight compounds that are challenging to clear with conventional hemodialysis [1,2], and it improves the elimination of PBUTs like indoxyl sulfate and p-cresyl sulfate [3,4,5]. The efficacy and clinical benefits of HDF are closely tied to the total convective volume or ultrafiltration delivered per session, a key indicator of the removal of these larger solutes [6,7,8,9]. Solute clearance in HDF relies on several factors: the filtration fraction (the ratio of ultrafiltered plasma water flow to blood flow rate), the blood flow rate, the sieving capacity of the filter, which is strongly influenced by blood flow rates, and the overall treatment duration [6,7]. As demonstrated by the recent CONVINCE study, achieving a threshold convective volume of 23 L, in addition to the net ultrafiltration volume, was essential to reducing all-cause mortality by 23% [10]. Further confirmed by an updated individual patient data meta-analysis involving more than 4000 patients, hemodiafiltration is associated with a reduction in relative risk of death both in all-cause and cardiovascular origins in an almost linear fashion with convective volume as more is better [11,12,13,14]. The clinical and biological benefits of high-volume hemodiafiltration in kidney care have been clearly highlighted in a recent comprehensive review [15].

Central to this process is the hemodialyzer membrane, which acts as a selective barrier, allowing uremic toxins to pass through while retaining essential proteins, including albumin [16,17,18]. The effectiveness of HDF also depends on patient-specific blood factors (e.g., hematocrit and protein concentration), transmembrane pressure management (either manual or automated), and operational conditions such as blood flow rate, substitution modality (post- versus pre-dilution), anticoagulation, and net ultrafiltration [7,19]. Early dialysis membranes, composed of cellulose, were limited in their capacity to clear larger molecules and frequently triggered inflammatory responses due to poor hemocompatibility and low hydraulic permeability [18]. Advances in materials and fiber-spinning technology have led to the development of synthetic polymer membranes, such as polysulfone, polyethersulfone, and polyacrylonitrile, with fibers that have finely tuned pore size distributions, offering improved clearance and a better hemocompatibility profile [20,21]. The design and quality-controlled manufacturing of these membranes are critical to achieve an optimal balance between effective toxin removal and minimal loss of albumin. Middle Cut-Off (MCO) and super-high-flux membranes with larger pores enhance middle-molecule clearance in hemodialysis but may increase the risk of albumin loss when high ultrafiltration flow is imposed [22,23,24]. Moreover, contact between blood and the membrane triggers hemoincompatibility reactions, including the activation of clotting, complement, and bradykinin cascades, as well as circulating cell stimulation, potentially leading to systemic inflammatory responses and contributing to organ damage [25]. The formation of a secondary protein layer on the membrane further alters filtration dynamics, affecting clearance efficiency and increasing transmembrane pressures [1]. Automated management of these variables through advanced, proprietary features of modern hemodiafiltration machines is essential for maintaining patient safety and ensuring treatment efficacy. Additional challenges, such as maintaining stable transmembrane pressures, preventing back-filtration, and controlling electrolyte balance, can further complicate high-volume HDF, making both membrane selection and the interaction between dialyzer and HDF machine crucial. While all of these factors are important, the present manuscript focuses specifically on the role of the dialysis membrane. The ideal membrane must meet strict criteria in terms of permeability, hemocompatibility, and stability under high convective volumes, thereby establishing it as a key component in advancing HDF as mainstream kidney replacement therapy. In this narrative review, we analyze the main components involved in and supporting mass transfer processes in hemodialysis (HD) and hemodiafiltration (HDF) from both physiological and engineering perspectives. The objective is to bridge mechanistic understanding with clinical practice and to provide clinicians with a translational framework that supports improved treatment strategies, facilitates the achievement of therapeutic targets, enhances efficiency, contributes to better patient outcomes and survival, and ultimately supports more sustainable and cost-effective dialysis care.

## 2. Hemodialysis Membrane and Solute Transfer

### 2.1. Membrane Material

In the early years of dialysis, membranes were primarily produced from regenerated cellulose. The characteristics of the cellulose-based membrane were a comparably low hydraulic permeability or ultrafiltration flow rate for plasma water [16,18]. Dialysis machines could regulate the weight loss of patients simply by means of transmembrane pressure control. Symmetric membrane walls with a thickness of 6–8 µm entailed short diffusive distances and proved to be efficient in diffusive clearance. While clearances of small molecules, such as urea and creatinine, were satisfactory, middle-molecular substances, such as, e.g., ß2-microglobulin, could not be removed in an adequate manner.

With the appearance of membranes spun from synthetic materials, different elimination characteristics were achieved, or were the consequence of novel production processes was much harder to control. Pore size spectra covered a much higher range, and filtration flow rates were considerably increased [26]. As the flow resistance of a membrane pore decreases by a power of four (law of Hagen–Poiseuille), doubling the pore diameter meant a sixteen-fold higher filtration rate. Care had to be given to the aspect of potentially excessive plasma water loss during the dialysis treatment, which was leading to the development of dialysis machines with dialysate volume balancing systems and a tight control of ultrafiltration volumes. The ultrafiltration capacity of the membranes beyond the volume needed for fluid status control of dialysis patients has offered the prospect of additional filtration volumes in the hemodiafiltration modality.

Various polymers have been used to produce dialysis membranes. The first materials successfully used were acrylonitrile (Rhone Poulenc), polyacrylonitrile (Asahi), polysulfone (Amicon), polyamide (Gambro), and polymethylmethacrylate (PMMA, Teijin). However, membrane characteristics cannot be derived from the polymer alone since membrane production, including sterilization, has a significant effect on the final membrane morphology and pore size distribution [27]. Furthermore, optimal hydrophilicity distribution is often reached by adding polar polymers [28,29]. However, suitability depends on transmembrane resistance during the treatment as well, which seems to be more critical in PMMA and triacetate membranes [30].

### 2.2. Membrane Hemocompatibility

A basic prerequisite of dialysis membranes is the hemocompatibility of the membrane material, as well as the potting material separating the blood from the dialysate compartment and the materials of the dialyzer housing. Low coagulation activation and low stimulation of active immune response (e.g., complement, phase contact and bradykinin systems) are key for the use in chronic kidney replacement therapy [25].

The development of new polymer compounds and membrane surface treatments has considerably reduced blood–membrane interaction reactions. In addition, modern automated production processes and production monitoring by improved laboratory equipment have continuously enhanced dialyzer quality. The migration of chemicals and release of particles have been reduced to extremely low levels, further minimizing immune system stimulation.

Considering the potential risks posed by dialysis fluid contaminants due to back-diffusion and back-filtration, synthetic high flux membranes achieve excellent retention of dialysate contaminants (e.g., bacteria, virus, endotoxins, by-products and organic/inorganic particles). This safety barrier of high-flux dialyzers, combined with point-of-use sterilizing ultrafilters in the dialysis machine’s hydraulic system for the preparation of ultrapure dialysis fluid or online preparation of sterile substitution fluid, minimizes inflammatory and immune system responses caused by dialysis fluid contaminants.

MCO membranes have larger pores than high-flux membranes, which facilitate, through internal filtration phenomena, better removal of larger middle molecules by hemodialysis. But on the other side, the more open structure of the membrane could enhance the transfer of pyrogens like endotoxins and other bacterial contaminants into the patient’s blood, which can be present in non-pyrogen-free dialysis fluid. They are not suitable for HDF treatments because they exhibit significantly higher permeability to albumin and plasma proteins compared with standard high-flux membranes when exposed to high transmembrane pressure, resulting in clinically unacceptable protein losses.

### 2.3. Membrane Permeability: Pore Size Selectivity and Distribution

The total filtration flow rate across the dialyzer membrane, normalized for the applied transmembrane pressure, gives the ultrafiltration coefficient (*K_UF_*). It categorizes the membranes into low, medium, or high hydraulic permeability of water. However, the more relevant characteristic of low and middle molecular elimination performance cannot be derived from the *K_UF_*.

The steepness of the cut-off curve can be utilized as an essential performance parameter for the efficacy of high-flux membranes regarding the removal capacity of middle molecules. It is based on the sieving characteristic of the membrane and is quantified by the sieving coefficient *S*, which is defined as the ratio of the solute concentration in filtrate *C_f_* and that in feed solution *C_in_*:

(1) $$S=\frac{C_{f}}{C_{in}}$$

**Figure 1:**
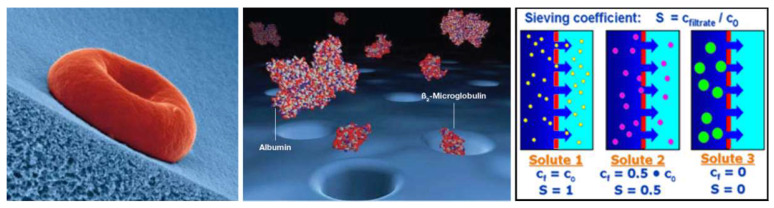
Size exclusion and sieving coefficient of dialysis membranes. (**Left**): comparison of the size of a red blood cell and the porous structure of the membrane. (**Middle**): small proteins (ß2m) fit into the membrane pores, but not large proteins (albumin). (**Right**): sieving coefficients of a membrane with homogeneous pore sizes for different solute dimensions (blue arrows indicate filtration flow).

Figure 1 depicts the principle of size exclusion of solutes by sieving characteristics of dialysis membranes.

Sieving coefficients for the spectrum of relevant molecular substances give deeper insight into the elimination capacity for uremic solutes. Typically shown in semi-logarithmic graphs, the sieving coefficients of high flux membranes stay at a value of 1, or a probability of 100%, to cross the membrane for substances in the low molecular range. The molecular weight of proteins, whose sizes exceed the smallest pore sizes, marks the onset of the cut-off region onset (MWRO) at sieving coefficient S = 90% (10% retention). It starts in modern high-flux dialyzers at molecular masses of about 10 kDa or of ß_2_-microglobulin (ß2m, 11.8 kDa). The upper end of the cut-off region is indicated by the molecular weight of proteins, whose sizes do not exceed the biggest pores of the membrane (MWCO) at a sieving coefficient of S = 10% (90% retention) [31].

A steeper sieving curve (*SC*) and a narrower cut-off region are achieved with a tighter pore size distribution and can be characterized by the molecular weight ratio of MWRO and MWCO:

(2) $${SC}_{steepness}=\frac{MWRO\;(S=90\%)}{MWCO\;(S=10\%)}$$

Beyond 60.000 Da, typically the size of albumin (HSA, 67 kDa), the sieving coefficients reach values below one percent to limit the loss of albumin and other large proteins, especially under hemodiafiltration conditions. The sieving characteristic is adjusted to that of healthy kidneys in this molecular range.

**Figure 2:**
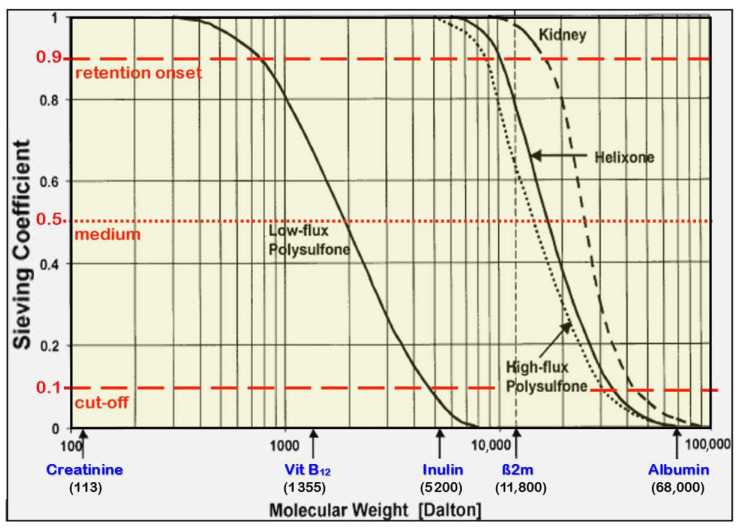
Sieving coefficient of low- and high-flux polysulfone membranes and of the kidney as a function of the molecular weight.

In case of low flux membranes, the molecular weight cut-off value (MWCO with 90% retention) is much smaller, i.e., only low molecular substances can be effectively eliminated due to the small pore sizes (see Figure 2).

In order to approach the entire sieving characteristic of the kidney, the sieving curves of the membranes would have to be much steeper. However, nowadays, membranes are limited by technical constraints in serial production.

The precipitation of the membrane by phase inversion is a stochastic process that generates broader pore size distributions with larger mean pore sizes. Consequently, MCO membranes usually have broader pore size distributions than high-flux dialyzers and have flatter cut-off sieving curves, and not the other way round, as often depicted.

### 2.4. Solute Membrane Transfer Mechanisms in HDF

#### 2.4.1. Solute Transfer by Diffusion and by Convection

Diffusion is the movement of solutes from a region with a higher concentration to a region with a lower concentration [32]. The concentration gradient *dC/dx* drives the diffusive solute flux. The thermal energy of the solution powers the motion, and the speed of the diffusion depends, therefore, on the temperature. Diffusion is a microscopic process that is demonstrated by the Brownian motion or random walk of small particles. Fick’s law of diffusion describes the flux of the solutes dn/dt driven by diffusion (in 1 dimension):

(3) $${\left(\frac{dn}{dt}\right)}_{diff}=-A*D*\frac{dC}{dx}$$

with

(dn/dt)_diff_: Diffusive flux of solute;
A: Area of membrane pores, through which the flux can penetrate;
D: Diffusion coefficient of solute;
DC/dx: Concentration gradient.

In dialysis, the plasma concentration *C_p_* of a solute is separated by the membrane from the dialysate concentration *C_d_*. The wall thickness of the membrane defines its distance *dx* = *s*. This gives an approximation of the gradient:

$${\left(\frac{dn}{dt}\right)}_{diff}=A*D*\frac{C_{p}-C_{d}}{s}=\frac{D}{s}*A*\left(C_{p}-C_{d}\right)$$

The diffusive flux is positive when the concentration in plasma is higher than in dialysate. This generates a solute transport from plasma to dialysate. With the definition of the permeability *k*_0_,

(4) $$k_{0}:=\frac{D}{s}$$

and the definition of the mass transfer coefficient *k*_0_*A* is

(5) $$k_{0}A:=k_{0}*A$$

which follows the important result for the diffusive mass transport:

(6) $${\left(\frac{dn}{dt}\right)}_{diff}=k_{0}A*\left(C_{p}-C_{d}\right)$$

The mass transfer coefficient *k*_0_*A* of dialyzer membranes is usually experimentally determined, considering the number and actual geometry of the pores, and that diffusion in porous media is slower than in the bulk of the solvent.

Convection is the solute transfer by the bulk movement of the solvent. The solute molecules are extremely small particles that are dragged along and moved in the same direction as the flow stream. The convective flux is given by the following:

$${\left(\frac{dn}{dt}\right)}_{con}=\frac{dn}{dV}*\frac{dV}{dt}=C*Q$$

or

(7) $${\left(\frac{dn}{dt}\right)}_{con}=C_{p}*Q_{f}$$

with

(dn/dt)_con_: Convective flux of solute;
C_p_: Plasma concentration of solute;
Q_f_: Filtration flow rate.

The ratio of the convective and diffusive fluxes gives

$$\frac{{\left(dn/dt\right)}_{con}}{{\left(dn/dt\right)}_{diff}}=\frac{Q_{f}*C_{p}}{k_{0}A*\left(C_{p}-C_{d}\right)}$$

Neglecting the dialysate concentration C_d_ results in

(8) $$\frac{{\left(dn/dt\right)}_{con}}{{\left(dn/dt\right)}_{diff}}\approx \frac{Q_{f}}{k_{0}A}=Pe$$

The ratio of the fluxes is identical to the Peclet number *Pe*. It represents the criterion that flux is more efficient in a clinical setting. When

- *Pe* < 1 (*Q_f_* < *k*_0_*A*): Diffusive flux is predominant;
- *Pe* > 1 (*Q_f_* > *k*_0_*A*): Convective flux is predominant;
- *Pe* ≈ 1 (*k*_0_*A* ≈ *Q_f_*): Diffusive and convective flux are equally potent.

**Figure 3:**
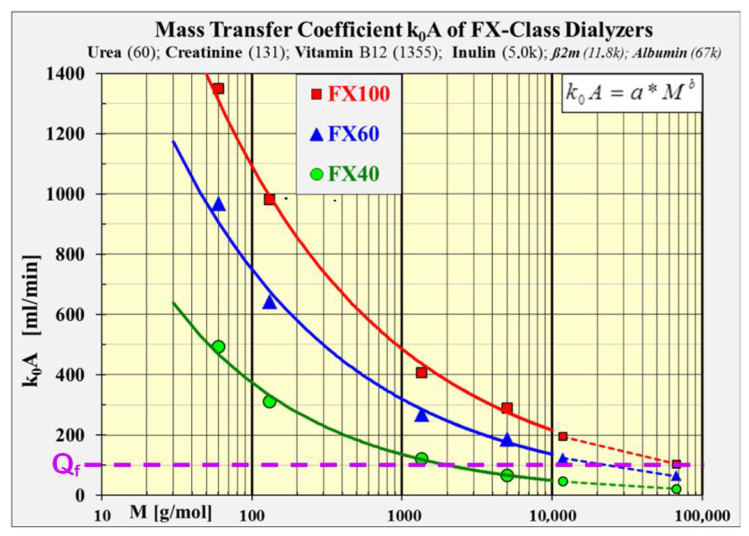
Mass transfer coefficients of dialyzers with identical capillaries as a function of the molecular mass M, measured in saline solutions. Membrane areas: FX40: 0.6 m^2^; FX60: 1.4 m^2^; FX100: 2.2 m^2^. Dotted lines: Extrapolation of the fit function in the molecular range from ß2m to albumin. Pink line: Filtration rate of Q_f_ = 100 mL/min.

Dialyzers of a model series are usually assembled with the same kind of capillaries, which have identical permeabilities *k*_0_. Their mass transfer coefficient *k*_0_*A* varies with the number of installed capillaries and is thus proportional to the membrane surface A of the dialyzer. Figure 3 shows the conspicuous impact depicted for a small (FX40), a medium (FX60) and a large (FX100) area dialyzer.

The thermodynamic law of equipartition of energy states that, in thermal equilibrium, the energy is shared equally among all molecules. They have the same translational kinetic energy, which means that the higher their molecular masses are the slower is the motion of the molecules. This hampers the diffusive mobility and leads to declining diffusion and mass transfer coefficients with higher molecular masses. Figure 3 shows the steady decline of the *k*_0_*A* for heavier molecules.

The *k*_0_*A* fit functions of the three dialyzers are extrapolated to the molecular mass of albumin (M = 67 kDa).

The traction of the solvent on solute molecules does not depend on their masses. Convective solute transfer is independent of molecular weight and depends solely on solvent drag as filtration rate (*Q_f_*) and the sieving coefficient. Therefore, the filtration rate *Q_f_* is depicted in Figure 3 as a horizontal line, indicating a constant mass transfer characteristic at a typical filtration rate of *Q_f_* = 100 mL/min.

It is intriguing that diffusion is a much more potent transport vehicle than convection for low molecular weight solutes up to M = 1000 Da. The diffusive clearance for urea (60 Da) or creatinine (131 Da) is a magnitude superior to that of convection. The gain of clearance is small in this molecular class when switching from HD to HDF treatment. The dialysate flow rate can be reduced in HDF by the substitution flow rate, because it does not matter for the clearance of these small molecules whether the dialysis fluid enters the dialyzer as dialysate through the inlet or as filtrate through the membrane. This means that when in HDF, the total flow rate of dialysate and substitution fluid equals the dialysate flow rate in HD, and at least the same removal of small molecules is achieved. Usually, the urea clearance in HDF exceeds that in HD by about 5–10% under this condition [33]. Therefore, in modern HDF treatments, there is no difference in the consumption of water, energy, and dialysis concentrates compared to HD treatments [2].

Diffusion can compete in medium-sized dialyzers (FX60) with convection up to the middle molecular weight range of about 20 kDa. In large-sized dialyzers (FX100), this range is expanded up to the molecular weight of albumin (67 kDa). The performance of free diffusion is amazing. But the solute transfer is hampered in modern dialyzers above a molecular mass of about 10 kDa because the molecules cannot pass the smaller pores of the membrane anymore due to their size. It has a detrimental impact on the diffusive as well as the convective transport mechanisms when the molecular size is comparable to the pore diameter. But as diffusion is characterized by a random-walk motion of the molecules, it is more restricted than in convection, in which the solutes are dragged and carried along with the solvent flow. With increasing molecular mass, the efficacy advantage tilts to the convective transport.

#### 2.4.2. Solute Transfer Associated with Back Transfer (Diffusion and Filtration) Mechanisms: Benefits and Risks

The solute transfer on the membrane is not a one-way street. If the concentration on the dialysate side is higher than on the blood side, the substance is transferred by diffusion with the identical mass transfer coefficient k_0_A from dialysate into blood (back diffusion). The same applies to convective transport, when the filtration flux (back filtration) is reversed by negative pressure differences across the membrane in hollow-fiber dialyzers used with HD machines equipped with fluid-balancing systems [34,35,36].

Both on the blood and the dialysate side of the dialyzer, pressure gradients are present along the capillaries. They are established by the flow resistances *R_b_* on the blood and R_d_ on the dialysate side of the dialyzer and the corresponding flow rates *Q_b_* and *Q_d_*. The flow resistance *R_b_* is usually the one with a greater value because of the higher viscosity of blood and the narrow flow path inside the lumen of the capillaries.

**Figure 4:**
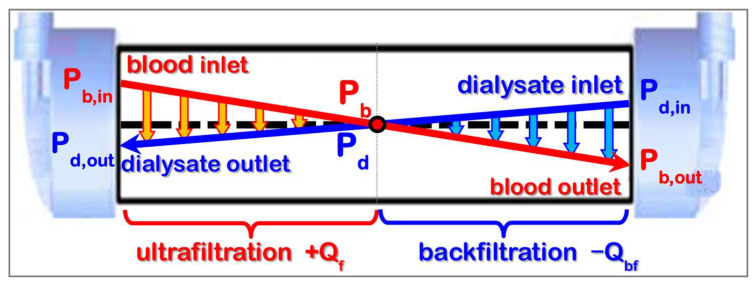
Pressure gradients along the capillaries drive the internal ultrafiltration. When the external ultrafiltration rate is small, *Q_uf_* ≈ 0, forward *Q_f_* and backward *Q_bf_* filtration rates are equal (fluid volumes are balanced by the hydraulics). Red line: Pressure on the blood side. Blue line: Pressure on the dialysate side (counter flow). Yellow Arrows: Local forward filtration rate. Blue Arrows: Local backfiltration rate.

Figure 4 shows the course of the local pressures inside the hollow-fiber dialyzer. It is typical for high-flux membranes in HD and for low-flux membranes with small ultrafiltration rates, because the transmembrane pressures are very low (*TMP* ≈ 0). At the blood inlet (dialysate outlet), there is higher pressure on the blood side, generating a forward ultrafiltration rate. The opposite conditions are present at the blood outlet (dialysate inlet), where lower pressure on the blood side generates a backward ultrafiltration rate. Forward and backward filtration rates are commensurate, as long as the *TMP* is small compared to the pressure drops along the capillaries.

The backfiltration flow rate increases with a higher ultrafiltration coefficient *K_uf_* and higher flow resistances on the blood *R_b_* and dialysate *R_d_* side. The backfiltration fraction *FF_bf_* can be estimated by Equation (9) when dialysate and blood flow rates are similar, and the oncotic pressure *π_b_* is negligible.

(9) $$FF_{bf}=\frac{Q_{bf}}{Q_{b}}\approx \frac{1}{8}*K_{uf}*\left(R_{b}+R_{d}\right)$$

For high-flux dialyzers with a narrow lumen diameter of 185 mm, the capillaries result in a backflow rate of about *Q_bf_* = 30 mL/min at a blood flow rate of *Q_b_* = 400 mL/min. *FF_bf_* is similar for all dialyzers of a model series with identical fibers because the *K_uf_* is proportional to and the flow resistor *R_b_* is inversely proportional to the number *N* of the capillaries (*R_d_* is usually negligible).

Standard dialysate, which is not ultrafiltered, is non-sterile and may contain endotoxins. The germs can normally not penetrate the membrane due to their size, which exceeds the pore sizes by far. But endotoxins can consist of lipopolysaccharides (LBS) (M_LBS_ = 10 kDa) and of lipid A (M_lipidA_ = 1.8 kDa). The Peclet number determines whether diffusion or convection dominates the transfer of endotoxins in the high-flux dialyzer FX60.

$$Pe_{lipidA}=\frac{Q_{fb}}{{\left(k_{0}A\right)}_{FX60;1.8kDa}}=\frac{30}{240}=\frac{1}{8}\;Pe_{LBS}=\frac{Q_{fb}}{{\left(k_{0}A\right)}_{FX60;10kDa}}=\frac{30}{120}=\frac{1}{4}$$

Both Peclet numbers indicate that diffusion is the more potent transfer mechanism for endotoxins. High flux membranes were conceived to enable the elimination of uremic solutes in the middle molecular range. As an inherent side effect, the necessity of larger pores led to much higher filtrate rates, which increase with the power of 4 of the pore size according to Hagen–Poiseuille’s law. Larger pores also increase the backflow rates, but the solute transfer by backfiltration remains small compared to the prevailing diffusion. Therefore, it is not effective to reduce the backfiltration rate in order to minimize the endotoxin load of the patient. The most efficient method is to apply ultrapure dialysate with accordingly low endotoxin counts (<0.03 EU/mL). As a result, both back-diffusion and backfiltration lose their relevance regarding the microbiological risk for the patient. The use of ultrapure dialysate is mandatory in high-flux dialysis when caring about the long-term well-being of the patients. This is addressed in the standard ISO 23500 series for water/concentrates for dialysis, dialysis fluid, and guidance for the preparation of fluids for hemodialysis and related therapies [37].

### 2.5. Conditioning of the Synthetic Membrane by a Protein Layer: Mechanisms and Implications

#### 2.5.1. Tightly Fixed Attached Secondary Membrane—Vroman Effect

Blood is a very intricate fluid if it is taken to an extracorporeal system. When any artificial material is in contact with blood, proteins from the blood plasma are adsorbed onto the material surface. According to the Vroman effect, these proteins may be displaced by a series of other plasma proteins, all within a time scale of minutes. The sequence of adsorption runs from proteins present at higher concentrations in normal plasma to proteins of lower concentrations, but with high binding affinities:

- Albumin;
- Immunoglobulins (IgG);
- Fibrinogen and fibronectin;
- Factor XII and HMWK.

In the end, a layer of plasma proteins covers the surface, which profoundly changes the characteristics of the artificial membrane. This protein layer sticks firmly to the basic membrane and cannot be removed even by intense rinsing along the capillaries or transmembrane flushing.

#### 2.5.2. Reduction in Ultrafiltration Coefficient (K_UF_) and Sieving Coefficient (SC)

**Figure 5:**
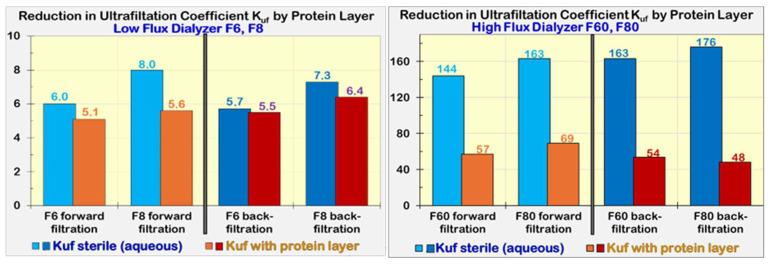
Ultrafiltration coefficients *K_uf_* measured in forward and in backward filtration for low-flux (**left**) and high-flux (**right**) dialyzers without (“sterile (aqueous)”) and with a protein layer. Back-flushing the membrane with saline solution did not restore the sterile *K_UF_*! [38].

Figure 5 depicts the ultrafiltration coefficients (K_UF_) of low-flux dialyzers, F6 and F8, and of high-flux dialyzers, F60 and F80. They were measured before (K_UF_ sterile (aqueous)) and after (K_UF_ with protein layer), when the membranes were covered with a plasma protein layer. When the ultrafiltration coefficients were determined in forward filtration (saline from blood to dialysate side), a minor reduction was found for the low-flux membranes, but a considerable change for the high-flux membranes. This indicates a strong flow restriction of water by the protein layer. When the ultrafiltration coefficients of the protein-coated membranes were determined in backward filtration (saline from dialysate to blood side), comparable reductions were found for low-flux and high-flux membranes as in forward filtration. This confirmed that the protein layer cannot be removed by rinsing back through the membrane.

**Figure 6:**
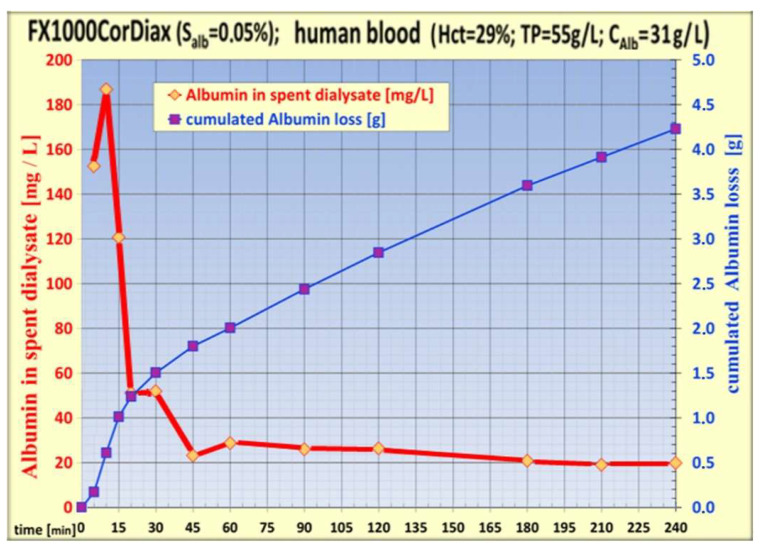
Temporal albumin loss into dialysate during a simulated post-dilution HDF treatment.

The temporal build-up of the protein layer in high-flux dialyzers can be monitored by the albumin loss into spent dialysate over time. When the membrane comes initially in contact with blood plasma, it has a high permeability even for large proteins like albumin, which implicates a comparably high albumin loss into dialysate (see Figure 6). Characteristically, during the first 20 min to 40 min, the albumin loss falls sharply and reaches a steady state with nearly constant loss until the treatment ends. The initial albumin peak counts for about 50% of the total albumin loss per treatment [39]. An important feature of the HDF machine lies in the way TMP is managed through dedicated algorithms, such as the proprietary AutoSub+ function (e.g., Fresenius Medical Care, Bad Homburg, G). This system tightly controls membrane protein layer formation by limiting high ultrafiltration flow during the initial phase of HDF, thereby preventing excessive albumin loss, which otherwise occurs predominantly within the first hour of treatment. This example illustrates the benefits of the interaction of membrane properties and HDF machine technology in optimizing HDF performance [1].

**Figure 7:**
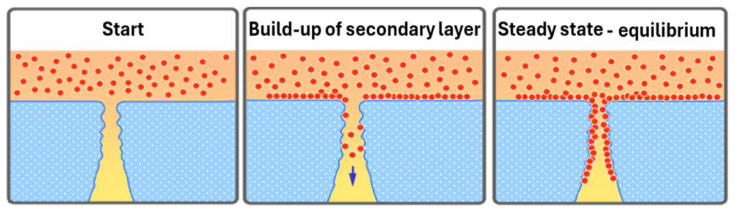
Model of secondary membrane build-up and blocking large molecules.

A plain model is depicted in Figure 7 showing the build-up of a protein layer blocking the passway of large molecules. The pristine membrane has a host of large pores whose diameters are still larger than the size of the relevant plasma proteins. This allows an easy protein transfer across the membrane into the spent dialysate, which explains the high initial protein loss. Over time, proteins stick to the inner surface of the capillaries and the pores, reducing their free diameter. Plasma proteins are now blocked or hampered from crossing the membrane. Once a complete mono-layer of proteins is established, the process of protein adsorption on the surface is finished. A steady state is reached in which the remaining protein transfer happens through pores being still big enough.

The build-up of the protein layer not only curtails the albumin transfer, but also facilitates the exchange of all solutes with molecular sizes in the cut-off region of the membrane. The cut-off region characterized by the sieving coefficient *S* is shifted to smaller protein sizes.

**Figure 8:**
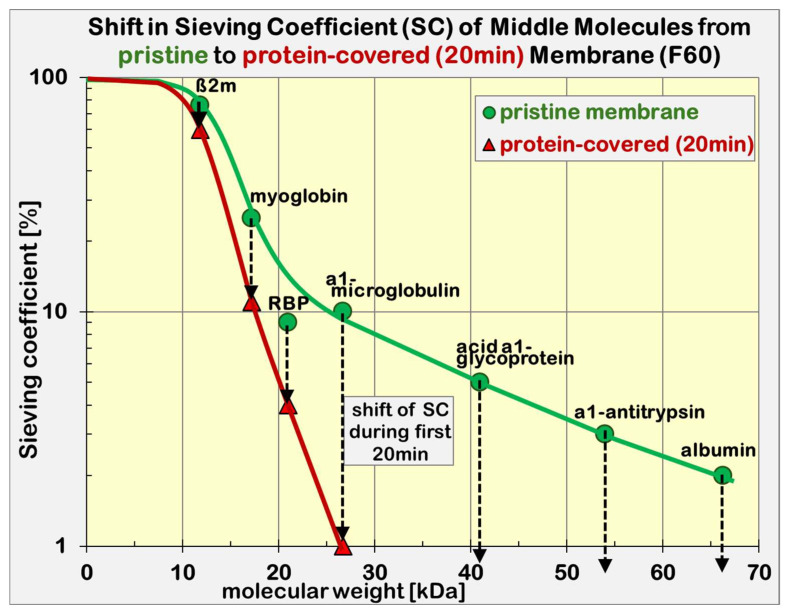
Sieving coefficient and protein layer formation on high flux polysulfone dialyzer F60 [40].

Figure 8 depicts the sieving coefficient of the high-flux dialyzer F60 in the cut-off range. It shows the sieving coefficient of the pristine membrane (green line) and the modified sieving coefficient after 20 min in contact with blood plasma (red line). The changes below the onset of the cut-off region (M < 10 kDa) are negligible. Inside the cut-off region, the relative change in its sieving coefficient S is more pronounced depending on the molecular weight.

#### 2.5.3. Clinical Consequences of the Protein Layer: Changes in Nominal Permeability

The initial reduction in the ultrafiltration coefficient of high-flux membranes is dramatically high (see Figure 5, right), but it has no relevant impact on the treatment. In HD with a rather high ultrafiltration rate of *Q_uf_* = 1000 mL/h, the *TMP* rises from 7 mmHg of the pristine membrane with a *K_UF_*_,1_ = 150 mL/h/mmHg to 20 mmHg with a resulting *K_UF_*_,2_ = 50 mL/h/mmHg due to the established protein layer. The *TMP* increase is negligible and is comparable to the tolerance of the *TMP* measurement in dialysis machines.

Even the colloid osmotic pressure (COP) of the blood, which adds to the TMP, is usually bigger than the *TMP* contribution caused by the dynamic pressure drop across high-flux membranes. This is also valid in post-dilution H(D)F treatments, as will be shown later.

The ultrafiltration coefficient K_UF_ of high-flux membranes has a low significance as a performance parameter for the removal of uremic solutes and should not be considered as a relevant criterion for the choice of a dialyzer.

The build-up of a protein layer curtails the exchange of all solutes with molecular sizes in the cut-off region of the filter from ß2m up to albumin. The sieving coefficient *S*, as specified in the dialyzer IFU, is valid only after conditioning of the membrane with plasma proteins. The standards EN1283 [41] and ISO 8637-1 [42] require as test fluid anticoagulated bovine or human blood plasma with a total protein concentration of *TP* = (60 ± 5) g/L and a plasma filtration fraction *FF_p_* of 20%. Surprisingly, the exposure time is not defined despite being a strong confounder.

For the *K_UF_* specification, the standards require bovine or human blood with a hematocrit of *hct* = (32 ± 2)% and a total protein concentration of *TP* = (60 ± 5)g/L as test medium.

In contrast to the *K_UF_*, the steepness of the cut-off curve (see Equation (2)) can be utilized as an essential performance parameter for the efficacy of high-flux membranes regarding the removal of middle molecules. A steeper sieving curve is achieved with a tighter pore size distribution, allowing better delimitation of solutes, which will be removed and which will be retained in plasma.

A further essential performance parameter is given by the sieving coefficient of albumin *S_alb_*_._ It serves as an upper limit for the removal of large middle molecules, so that the loss for proteins like albumin remains in a safe range.

Today, the plasma filtration fraction of *FF_p_* = 50% is common in high-volume HDF. As the apparent sieving coefficient usually increases with the filtration rate, the loss of albumin and proteins of comparable size can be quite different between test and treatment conditions.

The specification of the albumin sieving coefficient usually marks not the typical, but the upper limit value, which a dialyzer family may have. A specified value of *S_alb_* = 0.10% means that much smaller values like S_alb_ = 0.01% meet the specification as well. The albumin loss in a clinical trial with the same patients can differ by a factor of 10 when dialyzers from the same type, but different manufacturing batches are used.

Dialysis fluid or saline is used for the clearance determination of small molecules, like urea, creatinine, phosphate, vitamin B12, inulin, or small middle molecules, as their sieving coefficients are not hampered by the protein layer. But mass transfer coefficients, stated in dialyzer specifications, are determined with dialysate on the blood side as recommended in standards (ISO 1283, ISO 8637-1), and are remarkably higher than values measured with blood and in vivo. This is attributed to the viscosity of blood plasma, which is typically a factor of 1.84 higher than in dialysate [43].

#### 2.5.4. Antithrombotic Management of the Dialyzer and Extracorporeal Circuit: Unfractionated Heparin (UFH) Versus Low Molecular Weight Heparin (LMWH)

Unfractionated heparin (UFH) has been the anticoagulant of choice for many years, but it is replaced more and more by low-molecular-weight heparins (LMWHs). LMWHs are produced by depolymerization of UFH and have a typical mean molecular weight of about M = 4–6 kDa. They show a distribution in terms of chain length, molecular weight, and charge.

Whereas UFH is usually administered with an initial bolus followed by a constant infusion rate, LMWHs are administered as a single bolus at the start of the treatment. The anticoagulants are infused into the arterial blood line, upstream of the dialyzer. During the first dialyzer passage, a large part of LMWH is not yet bound to antithrombin due to the excess bolus concentration. This leads to significant LMWH losses (up to 20–30%) because, in this initial phase, the dialyzer membranes are not yet protein-coated, which renders them highly permeable for free heparin.

Although most LMWHs are currently recommended for administration in the arterial line, post-dialyzer administration into the venous line would avoid unnecessary losses of the LMWH dose into the dialysis fluid [44]. The optimal bolus would be administered into the arterial or venous needle before the patient is connected to the extracorporeal circuit.

If not compensated by the bolus dosage, the invisible loss of LMWH leads to a higher amount of residual blood in the dialyzer, falsely interpreted as a high coagulation activity of the dialyzer.

### 2.6. Potential Risks Associated with Dialyzer Sterilization Modalities

One crucial step in the production of dialyzers is the sterilization process. To deactivate microorganisms often means to damage materials as well. ETO gas sterilization can be classified as comparatively harmless in this respect; however, degassing requires time, and residual amounts of ETO migrating out of the potting material during dialysis have often led to patient reactions. Radiation with gamma or X-rays by either a gamma radiation or e-beam source are alternative methods that require careful evaluation of potential material damage. As sterilization is the final production step, breakdown products will remain inside the dialyzer and must be eliminated by the priming/rinsing process. Inline steam sterilized filters show low levels of any type of residues since the sterilization goes along with the active elimination of any production residues or migrating substances.

## 3. Mechanisms Occurring Within the Dialyzer Membrane During HDF: Understanding Internal Processes

HDF distinguishes itself from HD by the build-up of an additional secondary membrane on top of the protein layer inside the capillary, which is mobile depending on the blood flow rate and is modulated in thickness by the substitution rate. The following chapter tells the story of that modulable secondary membrane.

### 3.1. Transmembrane Pressure and Protein Polarization at the Membrane

The protein concentration on the membrane can be estimated by a simple model with dead-end filtration, i.e., for the sake of simplicity, there is no cross flow on the feed side of the dialyzer (*Q_b_* = 0). The focus is on the opposite effects of the filtrate flow building up the secondary membrane and the diffusion trying to remove it in order to level out the concentration gradient.

The build-up of secondary membrane is driven by convective flow rate *Q_f_*, which carries the protein flux dn_p_/dt to the dialyzer membrane:

(10) $${\left(\frac{dn_{p}}{dt}\right)}_{con}=C_{p}*Q_{f}$$

with

C_p_: Plasma protein concentration;
Q_f_: Filtration or substitution flow rate.

Large proteins cannot penetrate the membrane (*S* ≈ 0) but accumulate on the surface, establishing a concentration gradient inside the capillaries. This provokes a diffusive flux of proteins back into the plasma bulk, which can be described as follows:

(11) $${\left(\frac{dn_{p}}{dt}\right)}_{diff}=-A*D*\frac{dC_{p}}{dx}$$

The equilibrium is reached when the total flux (dn_p_/dt)_total_ of the diffusive and convective transport becomes zero:

$${\left(\frac{dn_{p}}{dt}\right)}_{total}={\left(\frac{dn_{p}}{dt}\right)}_{con}+{\left(\frac{dn_{p}}{dt}\right)}_{diff}=C_{p}*Q_{f}-A*D*\frac{dC_{p}}{dx}=0$$

The solution of this ordinary differential equation is given by

(12) $$C_{mem}=C_{bulk}*e^{\frac{Q_{f}*R}{A*D}}$$

with

C_mem_: Protein concentration on the membrane surface;
C_bulk_: Protein concentration in the bulk of the plasma;
R: Inner radius of the capillaries;
A: Membrane area;
D: Diffusion coefficient of protein.

The following example shows the extent of the protein polarization *C_mem_* at the membrane, which is determined by the balance between convective transport to the surface and diffusion back into the bulk solution.

Example: Ultrafiltration rate *Q_uf_* = 120 mL/min, radius of capillary *R* = 100 um, membrane area *A* = 2 m^2^, diffusion coefficient of albumin *D_alb_* = 6 × 10^−11^ m^2^/s.

Result: *C_mem_/C_bulk_* = 5.3.

This example under extreme conditions illustrates that the protein concentration at the membrane surface can be several times higher than the bulk concentration. Under treatment conditions with cross flow on the blood side, the protein polarization is smaller since proteins of that layer are conducted out of the capillaries together with the blood flow.

**Figure 9:**
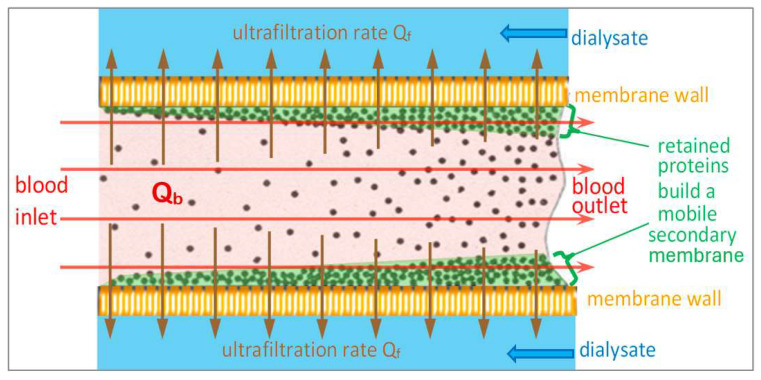
Flow and transport conditions inside a dialyzer capillary. Cross-filtration generates the mobile secondary layer on the membrane by retaining proteins. Red arrows: Blood flow. Blue arrows: Dialysate flow (counter current). Brown arrows: Ultrafiltration.

Figure 9 depicts more realistic flow conditions inside the dialyzer. The plasma proteins are flowing inside the blood flow stream *Q_b_* tangential to the membrane surface. The high ultrafiltration flow rate *Q_f_* perpendicular to the membrane surface conveys the proteins to the surface of the membrane. Depending on their size, they are retained and concentrated, still moving along the capillary as indicated by the red arrows. The protein polarization layer represents a moving secondary membrane of proteins whose concentration increases with distance from the blood inlet due to the increasing extracted UF volume.

The blood flow rate *Q_b_* is driven by a pressure gradient generated by the blood pump along the dialyzer capillaries. The higher shear stress produced by this flow entrains concentrated large molecules and keeps them moving to the blood outlet. When the ultrafiltration flow rate *Q_f_* stops, the whole polarization layer is washed out with a rinsing volume comparable to the dialyzer blood volume.

To gain a deeper insight into the characteristics of the secondary layer, a three-dimensional model of the dialyzer has been developed. The intriguing conclusion shows the true nature of the transmembrane pressure.

The model comprises three different flow domains—blood, inner volume of the membrane, dialysate—based on the Navier–Stokes law:

(13) $$\frac{\partial \vec{v}}{\partial t}+\left(\vec{v}\circ \nabla \right)\vec{v}=\frac{\eta }{\rho }*\Delta \vec{v}-\frac{1}{\rho }*\nabla P+\vec{g}$$

It is derived from the equilibrium of forces between inertial mass, internal friction, pressure gradients, and gravity on a finite volume element. The solution gives the local velocities $\vec{v}$ and the local pressures *P* inside the three domains of the dialyzer.

The motion and concentration build-up of the proteins (*C_p_*) and blood cells (*hct*) were simulated by the general transport equation:

(14) $$\frac{\partial C}{\partial t}+\vec{v}\circ \nabla C=D*\Delta C$$

It considers the convective and diffusive transport characteristics. The velocity $\vec{v}$ of the convection is obtained from the Navier–Stokes equation, as seen in Equation (13). However, the changed local concentrations, varying local viscosities *η* and densities *ρ*, have a direct influence on both velocity and pressure profiles. Therefore, Equations (13) and (14) were solved iteratively using the numerical CFD solver of ANSYS CFX 2020 R2.

The main result was the ultrafiltration characteristic of a dialyzer in HDF treatment. It describes the TMP necessary to push the ultrafiltration flow rate *Q_f_* across the membrane and could be very well described by the Starling equation [45]:

(15) $$Q_{uf}=K_{uf}*\left(TMP-\pi_{b,w}\right)$$

with

K_uf_: Ultrafiltration coefficient of the dialyzer with protein layer [mL/min/mmHg];
TMP: Transmembrane pressure [mmHg];
π_b,w_: Colloid osmotic pressure (COP) of the blood plasma proteins at the membrane wall [mmHg].

The COP at the membrane wall was calculated using the Landis–Pappenheimer equation [46,47]:

(16) $$\pi_{b}=2.1*C_{p}+0.16*C_{p}^{2}+0.009*C_{p}^{3}$$

with

π_b_: Colloid osmotic pressure (COP) of plasma proteins [mmHg];
C_p_: Total protein concentration in plasma [g/dL].

The amazing finding was that the colloid osmotic pressure *π_b_* of the plasma proteins in Equation (15) was sufficient to yield an excellent agreement between TMPs calculated by the model and observation, even at very high concentrations in the polarization layer.

The COP is a phenomenon at semi-permeable membranes through which the solvent (water) can pass, but not all solutes. At physiological plasma protein concentrations of about C_P_ = 7 g/dL, the COP has the standard value of π_b_ = 25 mmHg.

**Figure 10:**
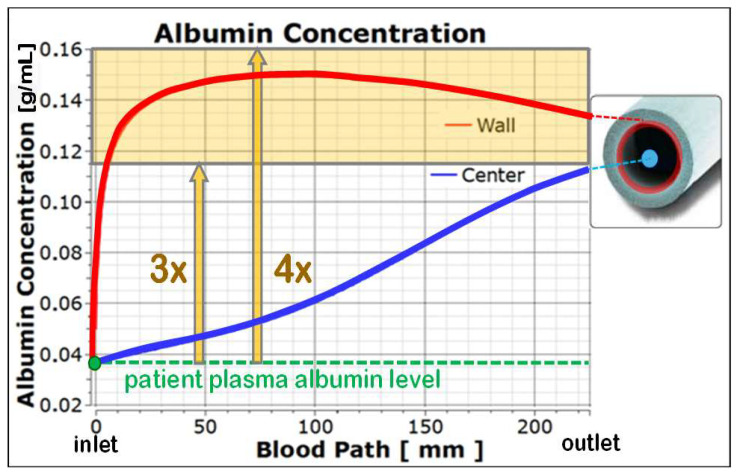

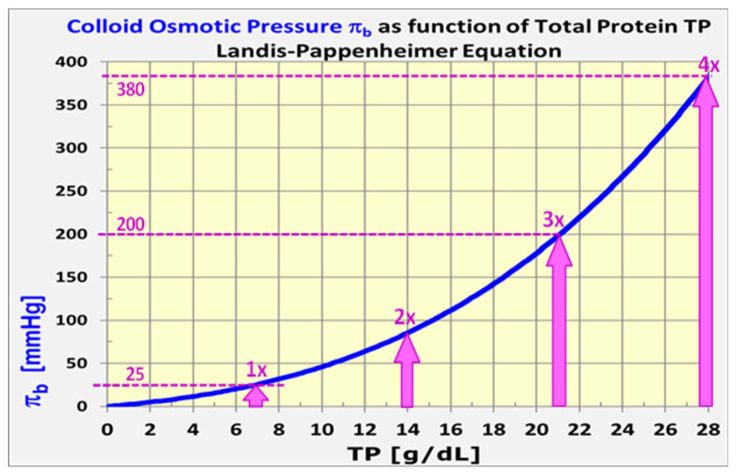
Colloid osmotic pressure and plasma protein concentrations inside the capillary. (Top): Plasma albumin concentrations along the capillary at the membrane (red) and on the middle axis of the capillary (blue). Simulation Parameters: *Q_b_* = 300 mL/min; *Q_uf_* = *Q_sub_* = 135 mL/min; *FF* = 45%; dialyzer: FX1000. (Bottom): Colloid osmotic pressure *π_b_* as a function of total plasma protein concentration *C_p_* according to Landis–Pappenheimer up to concentrations reached in protein polarization layers in post-HDF. Pink arrows indicate oncotic pressures at physiological plasma protein concentration 7 g/dL and at two-, three-, and four-fold levels.

Figure 10 (top) shows the calculated course of the albumin concentration along the capillary in high-volume-HDF post-dilution. The albumin concentration at the membrane rises steeply directly after blood inlet and reaches three- to four-fold bulk level after a short distance. These protein polarizations cause colloid osmotic pressure values π_b_ between 200 mmHg and 380 mmHg (see Figure 10 (bottom)), which equal typical TMP readings under HDF conditions. For the COP calculation, local concentrations of the accumulated proteins at the membrane *C_mem_* were applied instead of bulk protein concentrations *C_bulk_*.

Additionally to the colloid osmotic pressure *π_b_*, the dynamic pressure drop across the dialyzer membrane *∆P_mem_* contributes to the TMP [48].

(17) $$TMP=\Delta P_{mem}+\pi_{b}$$

The dynamic pressure drop *∆P_mem_* pushes the ultrafiltration fluid with the rate *Q_f_* through the pores of the membrane and is given by

(18) $$\Delta P_{mem}=Q_{f}/K_{uf}$$

**Figure 11:**
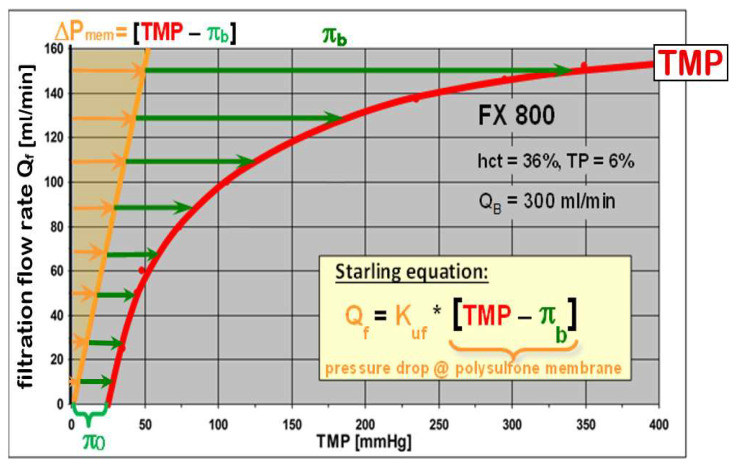
Ultrafiltration characteristic of high-flux membranes. Green arrows: The oncotic pressure *π_b_ = π*_0_ + *∆π_b_* of the plasma proteins at the membrane determines the behavior. Orange arrows: *∆P_mem_* is the pressure drop across the membrane caused by the filtration rate *Q_f_*.

The reciprocal value of the ultrafiltration coefficient 1/*K_uf_* corresponds to the flow resistance of the membrane *R_uf_*. Since the flow inside the membrane wall is laminar due to the very small dimensions of the pores, the pressure drop *∆P_mem_* increases proportionally with the ultrafiltration flow rate *Q_f_* (see Figure 11, yellow line). Forward filtration starts as soon as the *TMP* overcomes the COP *π*_0_ of the blood. With increasing filtration rates *Q_f_*, the protein polarization at the membrane surface and the associated COP gain ∆π_b_ progressively increases leading to a total COP *π*_b_ of

(19) $$\pi_{b}=\pi_{0}+\Delta \pi_{b}$$

The corresponding ultrafiltration characteristic *Q_f_* vs. *TMP* of an FX 800 dialyzer is shown in Figure 11. A zero net ultrafiltration rate *Q_f_* = 0 is achieved at a transmembrane pressure of *TMP* = π_0_ to counteract the oncotic pressure of blood plasma. The non-linear ultrafiltration characteristic of high-flux membranes is a consequence of the protein polarization and the resulting high oncotic pressure *π_b_* at the membrane (by Landis–Pappenheimer), which is always several times higher than the pressure drop *∆P_mem_* across the membrane and prevails the total transmembrane pressure *TMP*.

The viscosity inside the secondary membrane is elevated due to the protein polarization, and it generates an additional flow resistance for the plasma conveyed to the membrane by the filtrate rate. But the resulting pressure drop on top of the *TMP* is completely negligible, because the secondary membrane spreads across the entire membrane area (typically 2 m^2^) and has a very thin layer thickness of a few micrometers. Additionally, the velocity of filtration fluid v_f_ is extremely slow (e.g., *Q_f_* = 120 mL/min, *A* = 2 m^2^ => *v_f_* = *Q_f_*_/_*A* = 1 um/s).

### 3.2. Changes in Ultrafiltration Characteristics (K_UF_) During HDF Treatment

The ultrafiltration characteristic (*K_UF_*), which reflects changes in the hydraulic permeability of the membrane, is influenced by blood composition and by changes in blood volume (hemoconcentration) during treatment, resulting from net ultrafiltration, vascular refilling rate, and the patient’s weight loss to correct fluid overload. The prescribed weight loss causes blood thickening, and the increasing protein concentration leads to a higher colloid oncotic pressure (COP), resulting in a higher transmembrane pressure (*TMP*) at the same filtration rate.

**Figure 12:**
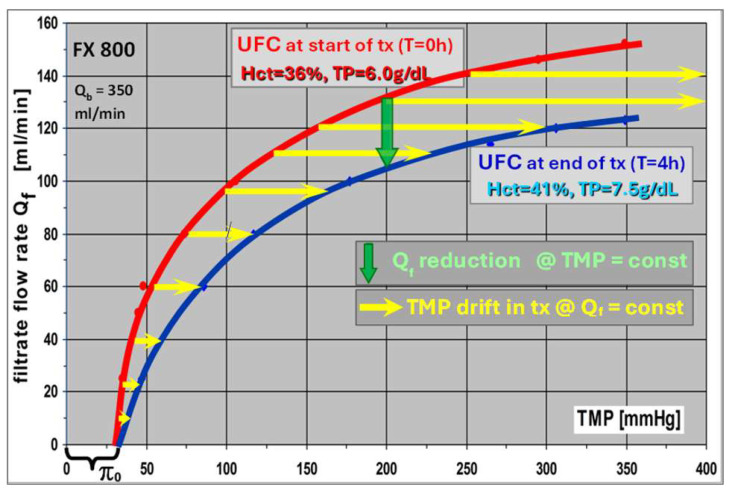
Ultrafiltration characteristic (*K_uf_*) of high-flux membranes for different blood compositions. Red line: *K_uf_* at *hct* = 36% and *TP* = 6.0 g/dL (start of treatment);- Blue line: *K_uf_* at *hct* = 41% and *TP* = 7.5 g/dL (with weight loss at end of treatment). Yellow arrows: *TMP* drifts at constant filtrate flow rates *Q_f_* during 4 h treatment. Green arrow: Reduction in filtrate flow rate *Q_f_* to keep a constant *TMP* of 200 mmHg.

The concomitant change in the *K_UF_* is shown in Figure 12 in a concrete example. The treatment starts at a *hct* = 36% and *TP* = 6 g/dL (*K_UF_*: red line). After 4 h treatment, the blood composition has changed to *hct* = 41% and *TP* = 7.5 g/dL (*K_UF_*: blue line). In HDF treatment with a constant exchange rate *Q_f_* (yellow arrows) of, e.g., 120 mL/min, the *TMP* reading is about 160 mmHg at the beginning (0 h) and about 300 mmHg at the end of the treatment (4 h). This is a shift of 140 mmHg in 4 h or 35 mmHg per hour.

Meanwhile, in HD, the *TMP* drift is negligible due to the small ultrafiltration rate *Q_uf_*, and it is clearly visible in HDF. Higher exchange rates *Q_f_* are intrinsically linked with a marked increase in the *TMP* drift, but at the same time, the convective clearance of middle molecules is far more efficient—the central purpose of HDF treatments. Unfortunately, very high exchange rates *Q_f_* can lead to excessively high *TMP* drifts during the treatment, which will trigger machine alarms. Therefore, the optimal convective clearance is reached with an exchange rate that ensures that the *TMP* stays just within the alarm limits.

HDF treatments with automatic *TMP* control are an alternative modality. The substitution rate *Q_f_* is controlled by the machine to keep a preset *TMP* value constant. For a chosen *TMP* of 200 mmHg in this example (see Figure 12, green arrow), the substitution rate *Q_f_* is reduced from initially 130 mL/min to 105 mL/min during the treatment. This more conservative mode prevents excessively high TMPs at the end of the treatment in all cases, which can cause additional severe albumin losses in this treatment phase.

For the clinician, the challenge remains in selecting the optimal prescription in manual mode, either a constant exchange rate (*Q_sub_*) or constant *TMP*, to achieve the best treatment efficiency.

### 3.3. Hemoconcentration Due to Excessive Transmembrane Pressure (TMP) and Ultrafiltration Rates

**Figure 13:**
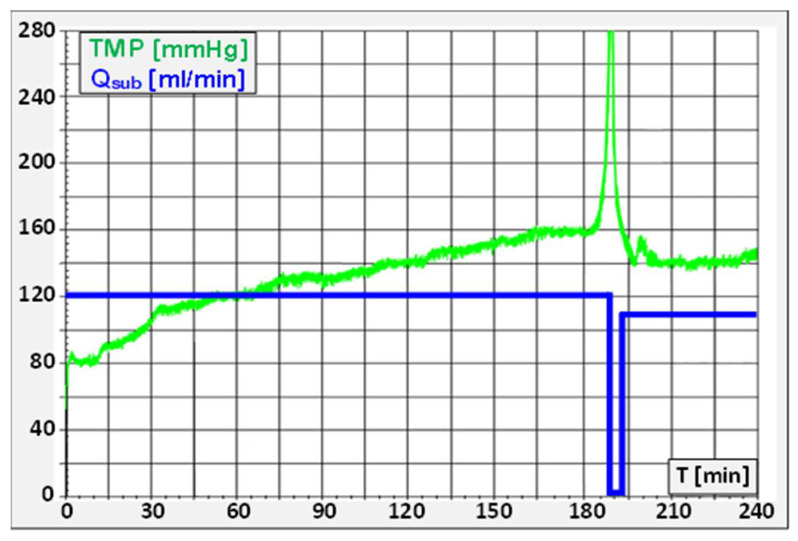
TMP drift in *HDF* post-dilution with constant substitution rate *Q_sub_*. Occurrence of an acute hemoconcentration after about 190 min. A short stop of substitution *Q_sub_* enables washing out of the polarization layer and restores the *TMP* at previous level.

As described above, high ultrafiltration rates may cause high protein polarization on the membrane. Acute hemoconcentration may occur if the protein flux arriving at the membrane via convection cannot be removed by diffusion and blood flow. This situation causes a sudden, steep increase in the protein polarization, which is marked by an abrupt and dramatic *TMP* surge, with characteristic rises of up to 100 mmHg/min (see Figure 13).

An additional factor contributing to excessive hemoconcentration is high net ultrafiltration with a low vascular refilling rate, reflecting substantial weight loss. This leads to a relative blood volume contraction of more than 10%, resulting in a marked increase in blood viscosity [49].

The recommended approach to managing this issue is to reduce or, preferably, temporarily stop the substitution rate *Q_sub_* for a few minutes. This action halts further protein polarization, allowing the concentrated blood within the dialyzer to be cleared with the ongoing blood flow *Q_b_*. As a result, the dialyzer’s flow and pressure characteristics are restored as soon as the polarization layer is washed out.

In all cases, it is essential to maintain a high blood flow rate to exert strong shear stress on the membrane. This shear stress effectively “scrubs” the membrane, reducing the thickness of the protein layer and preventing excessive protein polarization.

Acute hemoconcentration and clogging should not be confused with blood clotting in the extracorporeal circuit, although both conditions typically trigger *TMP* alarms as well as venous or arterial pressure alarms. The usual response to clotting involves increasing the heparin dose or, in some cases, discarding blood-filled lines and/or dialyzers. However, these actions do not resolve the underlying issue in cases of hemoconcentration and could result in significant blood loss, worsening the patient’s condition. Therefore, it is essential to accurately identify the cause of the alarms, as acute hemoconcentration generally occurs far more frequently than severe blood clotting.

The pursuit of the optimal high-volume-HDF treatment conditions leads to an unavoidable predicament: convective volumes of >23 L are required for highly efficient treatments, but lead to an increased risk of sudden hemoconcentration events. Additionally, the rapid pressure changes leave no time for the nurse to intervene adequately, and the occurrence of hemoconcentration events cannot be anticipated.

Consequently,

- The prescribed convective volume is not reached;
- The dialysis efficiency for middle molecules is reduced;
- The workload for the nurses is higher due to more interventions;
- Higher TMPs increase the albumin loss.

This implies that automatic control algorithms in the dialysis machines are needed, which determine the stress inside the dialyzers continuously and adjust the substitution rate accordingly. It must include a sensitive scouting monitor for the onset of hemoconcentration in combination with a rapid intervention procedure. In that way, the maximum exchange rates can be achieved under stable treatment conditions, avoiding intra-dialytic trouble.

### 3.4. Clinical Implications of HDF Use on Uremic Toxin Removal and Control

Several recent clinical trials [10,50,51,52,53] and meta-analyses [11,12,54] have shown that HDF treatments generate an improved patient outcome only when convective volumes larger than 23 L have been achieved. This observation raises an important question: What mechanistic and biological factors make high-volume HDF superior to conventional HDF or standard hemodialysis (HD)? In particular, which classes of uremic solutes are more effectively removed by this approach, and where might clinically relevant but still insufficiently characterized toxins reside?

**Figure 14:**
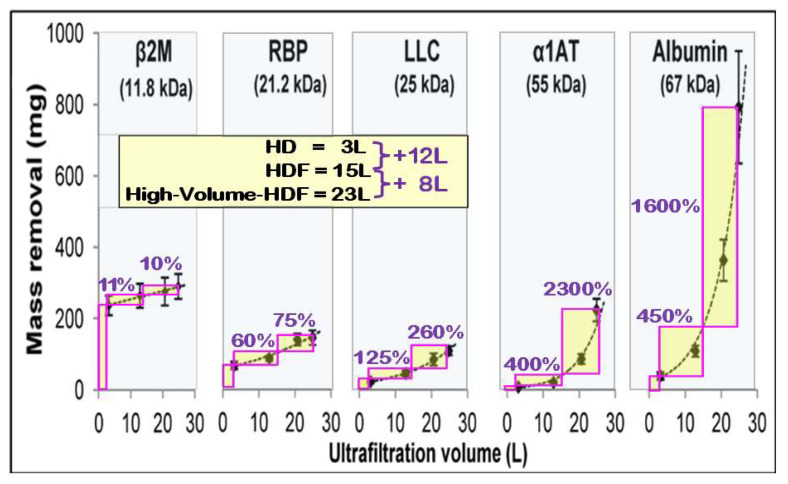
Increased mass removal of solutes with higher molecular weight by higher convective volumes. HD with weight loss of V_UF_ = 3 L, HDF with 15 L convective volume (12 L exchange volume + 3 L V_UF_); High-Volume-HDF with 23 L convective volume (20 L exchange volume + 3 L V_UF_) [56].

It is well documented that HDF only marginally improves the removal of small water-soluble solutes compared with HD. Its principal advantage lies in enhancing the clearance of middle and larger middle molecules in a convective dose-dependent manner. High-volume HDF specifically targets uremic toxins that are poorly removed by diffusion alone, including β_2_-microglobulin, fibroblast growth factor-23, parathyroid hormone, inflammatory cytokines, and protein-bound solutes such as indoxyl sulfate and p-cresyl sulfate. By addressing this broader solute spectrum, HDF overcomes key limitations of diffusion-based hemodialysis and contributes to improved clinical outcomes [55]. With modern high-performance dialyzers exhibiting high sieving coefficients for β_2_-microglobulin (S_β2m_ > 0.8), effective removal now extends to smaller middle molecules in the range of approximately 10–12 kDa. The incremental gain in mass removal when transitioning from HD to HDF, and subsequently to high-volume HDF, is therefore primarily observed within this molecular domain. For example, β_2_-microglobulin removal increases by roughly 10–15% when moving from HD to HDF and by an additional ~10% when high convective volumes are achieved, as illustrated in Figure 14.

Beyond 12 kDa, the removal efficiency improves dramatically with increasing molecular weight. The following effects can explain the observation:

1. Convection gets into narrow pores more efficiently than diffusion as a transport mechanism. One reason lies in the molecular mass of the proteins, which are comparable in size to the membrane pores. Another reason is the advantage of directed convective solute transport in narrow pores compared to an undirected diffusive motion.
2. Solutes with smaller sieving coefficients are retained largely by the membrane and form a dense protein layer. Their concentrations in the polarization layer increase exponentially with the filtration rate and cause the non-linear characteristic of the solute removal gain.
3. The high protein concentration on the membrane surface causes remarkably high oncotic pressure. It generates an osmotic flow from dialysate into the blood, which is several times higher than the substitution and weight-loss flow rates together, but it carries no solutes. The hydraulic pressure difference TMP pushes the equal flow rate back into the hydraulic to balance the fluid volumes. That directed flow rate transports solutes, enhancing the convective clearance.

With this removal behavior in mind, it is particularly important that the upper limit of the albumin sieving coefficient of high-flux membranes is very small, typically around 0.1%. This effectively limits solute removal in the higher molecular weight range, despite the marked relative concentration increase observed at higher convective volumes. In routine clinical conditions, albumin losses remain modest and generally have only a limited impact on patients’ circulating albumin levels.

This leads to the conclusion that, within the molecular weight range between β_2_-microglobulin (11.8 kDa) and albumin (67 kDa), high-volume HDF represents the most effective treatment modality currently available. Given that high-volume HDF has been associated with improved survival, it can be hypothesized that key uremic toxins contributing to adverse outcomes reside within this intermediate molecular domain. These likely include biologically active compounds such as cytokines, hormones, and adipokines, which are implicated in chronic inflammation, atherosclerosis, and cardiovascular disease, the principal drivers of morbidity and mortality in dialysis populations. Overall, these findings support the concept that the clinical benefit of high-volume HDF is closely linked to enhanced convective clearance of middle and larger uremic toxins. These solutes are increasingly recognized as major contributors to systemic inflammation, cardiovascular complications, and long-term morbidity in patients receiving maintenance dialysis.

### 3.5. Risk of Albumin Loss in High-Volume HDF

Albumin is not classified as a uremic solute, and the plasma levels in dialysis patients are often below physiological range. While albumin losses cannot be totally avoided in dialysis treatments, the removal should not reach unsustainable amounts. The scientific community has not yet established an agreement about the tolerable albumin loss per treatment. The discussed limits are very diverse and range from 3 g (in Europe and the Western World) up to 12 g (in Asia and Japan Region) per session.

**Figure 15:**
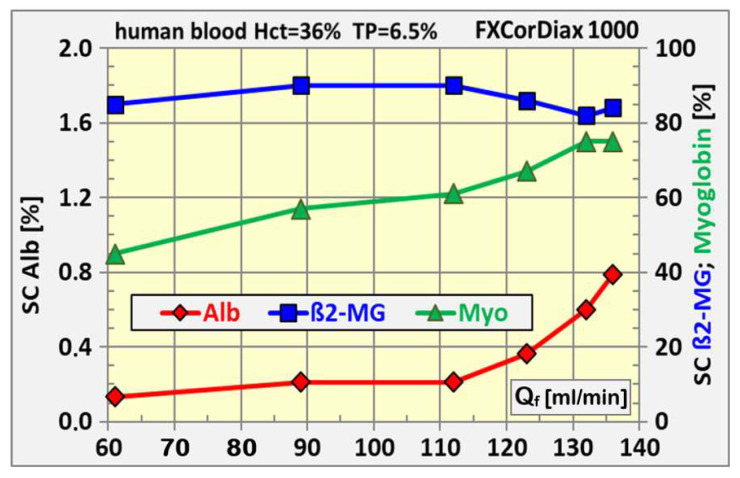
Sieving coefficient of different proteins as a function of filtration rate Q_f_. Note the high relative increase in S_alb_ at high filtration rates. Alb = albumin, ß2-MG = ß2-microglobulin, and Myo = myoglobin.

The main stimulus of the albumin loss is the substitution rate in conjunction with the inherent build-up of the secondary membrane and the sieving coefficient of the membrane. It must be considered that the sieving coefficients specified in the IFUs of dialyzers are measured at a plasma filtration fraction of *FF_p_* = 20%, according to the standards (ISO 1283, ISO 8637-1), but in high-volume HDF, much higher plasma filtration fractions like *FF_p_* = 50% are common and the apparent sieving coefficient under such clinical conditions can be several times higher, as shown in Figure 15 at higher exchange rates. This amplifies the albumin loss (see Figure 14).

When the filtration fraction FF_p_ is controlled by the dialysis machine to maximize the exchange volumes, the potential albumin loss must be kept in mind. The optimum middle molecule removal in combination with a safe albumin loss is accomplished by matching the cut-off characteristic of the filter and the algorithms of the dialysis machine to create an optimal comprehensive approach.

The ultrafiltration coefficient *K_UF_* is often taken as a criterion for choosing a high-flux dialyzer. Comparing the dialyzers ELISIO-H^TM^ and SOLACEA-H^TM^ (Table 1) shows that a higher ultrafiltration coefficient does not necessarily imply higher sieving coefficients. The disparity of sieving coefficients of both series is considerable, especially for large middle molecules. A rough estimation of the albumin losses m_alb_ in high-volume-HDF post-dilution for both filters yields

SOLACEA-H^TM^: *m_alb_* = *S_alb_* * *C_alb_* * *Q_f_* * *T* = 0.013 * 40 g/L * 120 mL/min * 4 h = 15.0 g
ELISIO-H^TM^: *m_alb_ = S_alb_* * *C_alb_* * *Q_f_* * *T* = 0.002 * 40 g/L * 120 mL/min * 4 h = 2.3 g

The significant difference between potentially critical and tolerable albumin losses could not be derived from the ultrafiltration coefficients.

### 3.6. Potential Clinical Implications of Albumin Losses

From a European perspective, albumin loss during dialysis is generally kept below 2–3 g per session (approximately 6–9 g per week), which is considered best practice in routine care. To date, there is no robust evidence demonstrating clinical benefit from albumin-leaking membranes or modalities designed to increase the removal of protein-bound uremic toxins, in contrast to some practices reported in Japan. The compensatory increase in hepatic albumin synthesis required to offset dialysis-related losses may be impaired in patients undergoing hemodialysis, particularly in the presence of chronic inflammation. Consequently, higher albumin losses during kidney replacement therapy, especially under conditions of high membrane stress, may be deleterious and should be regarded as a potential contributor to hypoalbuminemia. Moreover, serum albumin concentration is influenced by multiple factors, including inflammation, protein–energy wasting, and the burden of comorbidities common in dialysis populations. It remains one of the strongest prognostic biomarkers in this setting, reflecting both nutritional and inflammatory status and showing a strong association with morbidity and mortality. Finally, interpretation of serum albumin levels requires caution, as measurements may vary depending on the analytical method used (e.g., bromocresol green vs. bromocresol purple and other assays) and inter-laboratory variability. These methodological differences can significantly affect reported values and should be considered when using albumin as a clinical and research biomarker.

### 3.7. Substitution Modalities (Pre-, Post- and Mixed-Dilution HDF) and Their Implications

Various substitution modalities have been developed to overcome clinical limitations of post-dilution HDF. The substitution pump infuses ultrafiltered, sterile, and non-pyrogenic fresh dialysate from the volume-balancing system into the patient’s blood. If the infusion site is upstream of the dialyzer, the blood is pre-diluted, and if it is downstream of the dialyzer, it is post-diluted. In both cases, the balancing system retrieves the extracted volume across the dialyzer membrane as an additional filtration rate. The *TMP* is increased by lowering the hydraulic pressure until the filtration flow rate compensates for the extracted infusion rate.

In post-dilution plasma, water, together with all permeable ingredients, is removed. Thus, the filtration rate *Q_f_*_,*post*_ is equivalent to the convective clearance for large molecules. Typically, 50% of the plasma water flow rate *Q_pw_* can be exchanged. The blood cells and the residual proteins are concentrated accordingly inside the capillaries.

To achieve the same convective clearance in predilution, the infusion rate *Q_f_*_,*pre*_ must be about twice the value of post-dilution: *Q_f_*_,*pre*_ = 2 * *Q_f_*_,*post*_. It equals the plasma water flow *Q_pw_* and as in post-dilution about 50% of plasma water *Q_pw_* is exchanged. The uremic plasma solutes are distributed in the doubled plasma water volume, of which 50% is extracted in the dialyzer. In predilution treatments with high blood and high plasma water flow rates, respectively, the high fluid volumes exceed the filtration capacity of the membrane. In this case, post-dilution is superior, due to the smaller filtration rates of undiluted plasma water. Additionally, the concentration gradients of small molecules are not diluted, preserving a high diffusive clearance of small uremic solutes.

In treatments with low plasma water flow rates and large dialyzers, there is more filtration capacity than needed for the equivalent filtration rate in predilution. Under these conditions, pre-dilution is superior because more than 50% of the plasma solute can now be extracted by convection. Additionally, the frequency of hemoconcentration and the risk of unsustainable albumin losses are much less pronounced.

Patients with high hematocrit can be preferably treated with online-HDF pre-dilution as well, because of the lower plasma water flow fraction. In summary, the break-even between pre- and post-dilution depends on blood flow rate, hematocrit, and dialyzer surface.

In mixed-HDF, the total substitution flow rate is administered in a pre- (≈1/3 *Q_sub_*) and in a post-dilution (≈2/3 *Q_sub_*) fraction. Depending on the rheological constraints inside the dialyzer, the dialysis machine delivers the convective volumes more in pre- or post-dilution mode. The procedure always generates optimal treatment conditions adapted for each patient’s individual physiology.

### 3.8. Comparison of High-Volume External HDF and Internal HDF in MCO Membranes

The combination of medium cut-off (MCO) membranes with internal filtration (i.e., expanded HD and HDx) as well as high-volume hemodiafiltration (high-volume-HDF), focuses specifically on enhancing the removal of large middle molecules. In this range of molecular masses, both free diffusion and free convection contribute equally to solute transport. However, closer to the cut-off region of the membranes, convective transport is more efficient than diffusion since the mobility of large molecules in narrow pores is restricted. To avoid the reduced removal efficacy, the share of larger pores must be increased, which creates high demands on the control of the fiber-spinning process. Rather small shifts in the cut-off curve lead to significant changes in elimination efficiency. If the cut-off curve is not firmly reproducible in serial production, dialyzers from different lots will show a wide spread variability in reduction rates [22]. In addition, internal filtration depends strongly on blood flow, hemorheological conditions, and blood flow resistance, making it highly dependent on operational conditions. Furthermore, the MCO dialyzer functions as a “black box” with no meaningful or sample method to verify that the prescribed convective dose has actually been delivered, in contrast with online HDF.

This could be an explanation for the observation of different reduction rates of large middle molecules of two MCO dialyzers of the same family (Table 2) with different surface areas. The MCO dialyzer with the smaller area showed a higher reduction rate in the whole middle molecular range. Especially at the upper end, the differences were more pronounced, including the higher albumin loss.

High-volume-HDF can also be affected by variation in the average pore size, but the changes might be less profound, because convective transport and the built-up of an enhanced secondary protein layer can partially compensate for these differences. Furthermore, membranes with the intended use of HDF generally have a more conservative molecular cut-off.

Solute removal by convective transport is also present when MCO membranes are utilized, driven by internal filtration. The internal filtration fraction FF_bf_ can be estimated by Equation (9), because internal filtration and back-filtration are identical. As the MCO and high-flux dialyzers have comparable UFCs and lumen diameters of 185 mm, there is no difference in their respective FF_bf_. Typical backflow rates are 30 mL/min at a blood flow rate of 400 mL/min. In HD mode, the gain in large middle molecular clearance of MCO membranes is higher as a direct result of their larger sieving coefficients.

The mechanism of internal filtration depends strongly on the blood composition and viscosity of the individual patient. At the same time, the blood viscosity determines the flow characteristics through the hollow fiber as described by the fundamental law of Hagen–Poiseuille:

(20) $$R_{b}=\frac{\Delta P_{b}}{Q_{b}}=\frac{128}{\pi }*\frac{\mu *L}{D^{4}}$$

where

*Q_b_*: Blood flow rate;
Δ*P_b_*: Axial pressure drop;
*μ*: Blood viscosity;
*L*: Fiber length;
*D*: Diameter of the lumen.

An increase in flow resistance *R_b_* leads to a proportional increase in the axial pressure drop that is required to convey the blood flow through the capillaries. The hematocrit increases the viscosity *μ* of the blood [59], which is proportional to the flow resistance.

Blood with a low hematocrit contains a large plasma water fraction, which would allow a high convective removal. But the internal filtration fraction is low in MCO membranes due to the low blood viscosity and the concomitant little flow resistance *R_b_*. The passive control of the filtration rate leads to patient individual convective treatments, but not to optimized removal rates.

When patients with high hematocrits are treated, the resulting large flow resistance forces stronger internal filtration. Due to the limited amount of plasma water, the ensuing concentration of blood leads to even further filtration and may even cause a final blocking of the fibers by hemoconcentration.

In contrast, automatic algorithms in actively controlled High-Volume-HDF treatments sense the stress in the dialyzer and modulate the filtrate flow rate according to the present pressure and flow conditions. This yields a patient individual filtration rate with an optimal convective clearance, independent of the blood rheology of the patient.

### 3.9. Limitations of Mechanistic Modeling

We acknowledge that the mechanistic modeling approaches and underlying assumptions used in this section have inherent limitations. These analyses rely on simplified representations, including idealized membrane geometry and pore distribution, constant plasma composition, and steady-state conditions. They do not fully account for inter-patient variability, intra-patient temporal changes, differences in operating modes, or the effects of anticoagulation strategies and blood–material interactions during treatment.

Accordingly, the clinical translation of these findings should be interpreted with caution. The purpose of these models is to identify and illustrate the dominant transport mechanisms governing solute and fluid exchange in HD and HDF, rather than to reproduce the complexity of individual patient physiology or real-world treatment conditions. Integrating mechanistic insights with clinical judgment and patient-specific parameters remains essential when applying these concepts to routine practice.

## 4. Key Characteristics of the Optimal HDF Membrane

### 4.1. Optimal Membrane Characteristic

Although the detailed aspects of an optimal membrane for HDF application have been discussed in preceding chapters, they are described here again in a comprehensive summary.

(1) The steepness of the sieving coefficient curve.

High-flux dialysis membranes are characterized by the pore size distribution and the resulting function of the sieving coefficient curve with increasing molecular weight. The optimal characteristics would correspond to the natural example of the kidneys (biomimicry) because the elimination ratio of medium-sized proteins would match the physiological needs of a healthy subject. Like the steepness of the sieving curve of natural kidneys, the steepness represents an essential performance parameter for HDF dialyzers as well. Despite all improvements, the pore size distributions of modern membranes have achieved this ideal goal of the kidney function only partially.

(2) Restricting the albumin elimination.

The choice of the sieving coefficient of albumin by the upper end of the pore size distribution defines the second essential performance parameter. Additionally, the albumin elimination is modulated by the treatment conditions, i.e., the sieving coefficient must be specified according to the intended use. For high-volume-HDF in post-dilution, membranes must have a more conservative limit than for HD or HDF in pre-dilution.

(3) Lumen diameter and fiber length.

Since the convective filtrate volume is actively controlled by the dialysis machine, the internal filtration, depending on the dimensions of the fiber, is not required to achieve effective convective clearance. Therefore, there is no need to narrow the lumen diameter or increase the length of the fiber for HDF membranes. On the contrary, a larger lumen diameter should be selected in HDF, which reduces blood shear stress inside the dialyzer and mitigates internal filtration phenomena that may hinder ultrafiltration flow and solute clearance.

(4) Ultrafiltration coefficient KUF and plasma water permeability.

The primary development goal of high-flux membranes was to increase the pore sizes to allow an adequate middle molecular clearance. As a mere side effect, the larger pores caused a disproportional increase in the water permeability according to the law of Hagen–Poiseuille.

The comparably low filtration rates in post dilution cause only a small dynamic pressure drop, correlated to the ultrafiltration coefficient K_UF_, which leads to a rather small TMP contribution. Due to the high protein polarization, the TMP is dominated by colloid osmotic pressure. Therefore, water permeability or K_UF_ plays a minor role.

In pre-dilution, high filtration rates cause far higher dynamic pressure drops across the membrane, which predominates the TMP. Lower protein polarizations in pre-dilution lead to reduced colloid osmotic pressures. Hence, the water filtration capacity determined by the membrane area of a high-flux dialyzer is important.

### 4.2. Currently Available HDF Dialysis Membranes

The key characteristic of high-flux membranes used for Online-HDF consists of a steep cut-off characteristic and an albumin sieving coefficient restricting the albumin loss to an acceptable level (see chapter 0). The most suitable membrane materials meeting these requirements are synthetic copolymers, where polysulfone (PS) and polyethersulfone (PES), mixed with hydrophilic polyvinylpyrrolidone, play a predominant role (>90%). The remaining HDF membranes are made from other synthetic polymers, like polymethylmethacrylate (PMMA) or polyacrylonitrile (PAN).

## 5. The Gibbs–Donnan Effect and Its Implications for Electrolyte Balance in Post-Dilution Hemodiafiltration

### 5.1. Two Concerns of Unforeseeable Na Shifts in HDF Treatments

There are valid concerns that in HDF treatments, two additional electrolyte transfer mechanisms are induced, which could significantly alter the electrolyte balance. Sodium is of special interest due to its dominant plasma concentration.

Concern 1: Concentration Difference Between Plasma Water and Substitution Fluid

The sodium plasma water concentration is higher than in dialysate, when both sides are in equilibrium due to the Donnan effect. Nevertheless, there is no diffusive sodium transfer through the membrane in this case. In HDF, high convective volumes are drawn across the membrane with the higher plasma sodium concentration. The filtrate is replaced by a substitution fluid with the lower dialysate sodium concentration. The first concern is as follows: Could this concentration gradient, in combination with exchange volumes >23 L, induce a significant, non-indented sodium removal in contrast to HD?

Concern 2: Excessively High Donnan Factors by the Protein Polarization Layer

As the strength of the Donnan effect is determined by the negative charge density of the proteins present on the surface of the membrane, the emerging second concern has been that the sodium difference in equilibrium could also be increased with the protein concentration in the polarization layer (see Figure 10), totaling up to 28 g/dL. This could cause a Donnan factor of *F_D_*_,*HDF*_ = 0.80, and would require a dialysate sodium of *C_Na.d_* = 117 mmol/L to prevent diffusive Na flux into the blood. The standard dialysate sodium setting of *C_Na_*_,*d*_ = 138 mmol/L would be 21 mmol/L higher than the equilibrium concentration, leading to a high diffusive sodium flux into the patient. This has been the second concern.

In both cases, the dialysate sodium ought to be adjusted in order generate sodium flux over the membrane, which compensates for the convective loss and diffusive gain. The clinicians would have no means to deduce the appropriate dialysate sodium level, which would fit the momentary treatment conditions. In the end, this would be a huge obstacle for HDF in clinical routine and would require additional procedures for adequate electrolyte balancing.

However, the practice of routine high-volume-HDF therapy, experimental results, and theoretical models confirm that these fears and concerns can be refuted. The theoretical background of the Donnan effect and its impact on HDF will be explained in the following chapters [60].

### 5.2. Gibbs-Donnan Effect: Equilibrium of Diffusion and Coulomb Interaction

**Figure 16:**
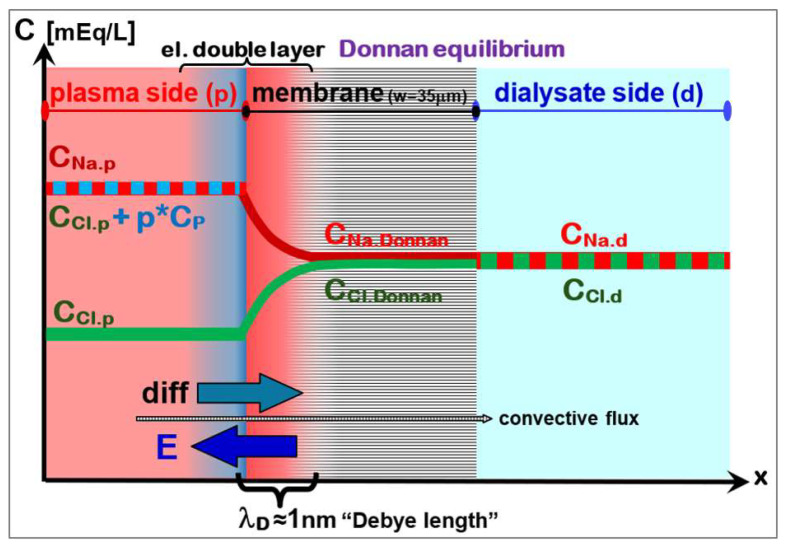
Concentration profiles (red line: Sodium and green line: Chloride) and electrolyte fluxes in the space-charged region under Donnan equilibrium. Arrows indicate diffusive (diff), electrophoretic (E), and convective fluxes of cations; arrow thickness depicts flux strength. Electro-neutrality condition in plasma (p): *C_Na_*_,*p*_ = *C_Cl_*_,*p*_ + *p* * *C_P_*; Index: Na: Sodium (z_Na_ = +1), Cl: Chloride (z_Cl_ = −1), P: Multiple charged proteins (z_P_ = −p).

The Donnan effect is a surface phenomenon on the interface between blood plasma and dialyzer membrane. The small cationic electrolytes (like Na) of blood plasma can easily enter the dialyzer membrane. Larger proteins, which are mostly negatively charged, cannot enter due to the size exclusion by the small pores. This generates a charge separation: While the membrane surface is positively charged, the plasma layer carries a negative charge. This space-charge region creates an electrical field, which retains cations (Na^+^, K^+^) and repels anions (Cl^−^, HCO_3_^−^) from the blood plasma (see Figure 16). The charge density difference between both sides generates an electrical field E and establishes the potential difference U in the double-layer, which can be described by the Nernst equation:

(21) $$-z_{i}*F*U=R*T*\ln\frac{C_{i,d}}{C_{i,p}}$$

with

U: Potential difference on the space-charge region [V].
z_i_: Charge number of ion i [1].
C_i,d_, C_i,p_: Concentration of ion i in dialysate (d) and in blood plasma water (p) in the Donnan equilibrium, i.e., no diffusive transfer happens [mol/L].
F: Faraday constant [96,485 As/mol].
R: Ideal gas constant [8.314 J/mol/K].
T: Temperature [K].

Separating Equation (21) into an ion-specific term (left, index i) and an ion-independent term (right) gives the definition of the Donnan factor *F_D_*:

(22) $$F_{D}:={\left(\frac{C_{i,d}}{C_{i,p}}\right)}^{\frac{1}{z_{i}}}=e^{-\frac{F*U}{R*T}}$$

The same Donnan factor F_D_ applies for all electrolytes i, but each ion has an individual concentration ratio *C_i_*_,*d*_/*C_i_*_,*p*_ depending on its charge number *z_i_*. The potential difference *U* is positive for the negatively charged plasma proteins, which gives Donnan factors of *F_D_* < 1.

On the semi-permeable dialyzer membrane, the concentrations *C_i_*_,*p*_ of cations (*z_i_* > 0), like sodium, are always higher in the blood plasma water than their equilibrium concentrations *C_i_*_,*d*_ on the dialysate side. With a typical Donnan factor in hemodialysis of *F_D_* = *C_Na_*_,*d*_/*C_Na_*_,*p*_ = 0.95 and a plasma sodium concentration of 145 mmol/L, the equilibrium sodium concentration in dialysate corresponds to 138 mmol/L, i.e., a sodium difference of 7 mmol/L prevents diffusive sodium transfer. The concentrations *C_i_*_,*p*_ of anions (*z_i_* < 0) are always lower in the blood plasma water than their equilibrium concentrations C_i.d_ on the dialysate side (e.g., *C_Cl_*_,*p*_ = 100 mmol/L → *C_Cl_*_,*d*_ = 105 mmol/L and *C_bic_*_,*p*_ = 24.0 mmol/L → *C_bic_*_,*d*_ = 25.3 mmol/L).

In complex mixtures like blood and dialysate, the Donnan factor F_D_ is calculated using the neutrality condition in both domains. Considering the cations sodium (Na), potassium (K), calcium (Ca), magnesium (Mg), and proteins (P) with the charge number z_p_. in the plasma water (p), it ensues

(23) $$F_{D}=\sqrt{1-\frac{\left|z_{P}\right|*C_{P}}{C_{Na,p}+C_{K,p}+2*\left(C_{Ca,p}+C_{Mg,p}\right)}}\approx \sqrt{1-\frac{\left|z_{P}\right|*C_{P}}{C_{Na,p}}}$$

Sodium is the dominating plasma electrolyte. The Donnan factor F_D_ depends only on the electrolyte concentrations in the plasma, where the proteins are located, and dialysate electrolytes have no impact.

#### 5.2.1. The Impact of High Convective Fluxes of HDF on the Gibbs-Donnan Effect

The distinctive characteristic of this space-charge region is its extremely narrow thickness of about the Debye length *λ_D_*, which is less than 1 nm at the ionic strength in plasma or in dialysate. The Debye length *λ_D_* is determined by the interaction of thermal and electrostatic energies with the ions. Nevertheless, the charged double layer is responsible for the difference in the sodium concentration between plasma (p) and dialysate (d) of typical *∆C_Na_* = *C_Na_*_,*p*_ − *C_Na_*_,*d*_ = 7 mmol/L. In detail, the Donnan effect can be described for HDF treatment as follows:

1. Because of the extraordinarily narrow thickness of the electrical double layer *∆x* = *λ_D_*, an extremely high sodium concentration gradient of *∆C_Na_/∆x* = 7 mmol/L/1 nm = 7000 mol/L/mm exists in the layer. That gradient causes a very high diffusive sodium flux of *∆N_Na_/∆t* = *D_Na_* * *A* * *∆C_Na_/∆x* (*D_Na_* = 1.9 × 10^−9^ m^2^/s; *A* = 1.6 m^2^):
  *∆N_Na_/∆t* = 1.9 × 10^−9^ m^2^/s * 1.6 m^2^ * 7000 mol/L/mm = 76,600 mol/h.
  The diffusive solute fluxes in the double-layer are exceptionally high.
2. The negatively charged plasma proteins cannot follow due to their size. A space-charge region is generated in which an electrical field E is established directing from the membrane to the blood compartment. The electrical field *E = U/∆x* generates an electrophoretic flux inside the double layer, which drives the cations back to the blood compartment and counterbalances their diffusive flux. The steady state is reached when both fluxes cancel each other out to zero. This is the Donnan equilibrium with characteristic electrolyte concentrations.
  When the electrolyte concentration in the dialysate is equal to the equilibrium concentration, no diffusive exchange occurs (see Figure 16).
3. An additional flux of electrolytes is conveyed in HDF by the convective ultrafiltration flow rate through the membrane. But even at high filtration rates *Q_f_* achieved in high-volume-HDF, this convective flux is very small compared to the diffusive flux in the space-charge region.
  **Example:** At a very high exchange rate of *Q_f_* = 120 mL/min, the convective sodium flux only reaches *∆N_Na_/∆t* = *C_Na_*_,*p*_ * *Q_f_* = 145 mmol/L * 120 mL/min = 1 mol/h.
  This convective sodium flux is about five orders of magnitude smaller than the diffusive sodium flux (and the equal electrophoretic counter-flux). It contributes only to a very weak perturbation of the fluxes created by the Donnan effect and is therefore negligible. The concentration ratio *C_i_*_,*d*_*/C_i_*_,*p*_ across the space-charge region, defined by the Donnan equilibrium, is not altered even with the additional convective flux achievable in HDF treatments.
4. When electrolytes are dragged along by a convective filtration rate Q_f_ inside the capillary lumen, the fluid has the plasma water bulk concentration, e.g., for sodium, C_Na,p_ = 145 mmol/L, until the onset of the double layer. Since the cations are partially repelled by the electrical field, their concentrations steadily fall along the flow path through the space-charge region. When the filtrate flow leaves the electrical double layer (λ_D_ ≈ 1 nm), the ion concentrations in are stripped down to the concentrations of the Donnan equilibrium [61], e.g., for sodium, C_Na,Donnan_ = 138 mmol/L.
5. In the case of anions, e.g., chloride with *C_Cl_*_,*p*_ = 100 mmol/L, the electrical field *E* attracts the negatively charged ions in the plasma and accelerates them in the space-charge region, which steadily increases their concentrations during the passage of the double layer up to the concentrations of the Donnan equilibrium, e.g., for chloride, *C_Cl_*_,*Donnan*_ = 105 mmol/L.
6. When the electrolytes leave the double layer, their concentrations are equal to the equilibrium concentrations determined by the Donnan effect, even at very high convective streams of plasma water penetrating the space-charge region in high-volume HDF.
7. The Donnan effect ensures that the total flux of all electrolytes reaching the dialysate side of the membrane is electrically neutral.

The concentration profile in the membrane induced by the Donnan effect is illustrated in Figure 16 for a plasma containing only negatively charged proteins (P^p−^), sodium (Na^+^) and chloride (Cl^−^) ions for simplicity. The concentrations of the electrolytes differ between the blood and dialysate sides, though both sides are in thermodynamic equilibrium. The dynamic equilibrium is established by the diffusive (Diff) and electrophoretic (E) ion fluxes, which are equally strong but moving in opposite directions. The concentration gradient in the double layer does not drive a net flux out of it.

When the dialysate concentration *C_i_*_,*d*_ of an electrolyte i is set to the equilibrium concentration of the Donnan effect *C_i_*_,*Donnan*_, no net diffusive flux occurs. Otherwise, e.g., when *C_i_*_,*d*_ is set equal to the plasma concentration, the concentration difference drives a diffusive flux (w: wall thickness of membrane, i: ion i) through the membrane:

∆*N_i_*_/_∆*t* = *D_i_* * *A* * (*C_i,Donnan_* − *C_i,d_*)/w (24)

The electrolytes in the ultrafiltration fluid have adopted the concentrations set by the Donnan-equilibrium after passage of the double layer. This means that convective volumes do not remove plasma water with plasma electrolyte concentrations *C_i_*_,*p*_, but with Donnan-equilibrium concentrations *C_i_*_,*Donnan*_. This leads to an accumulation of cations and a deprivation of anions in plasma. The plasma remains electrically neutral because the diffusive and convective fluxes carry no net charges through the membrane.

In summary, the convective flow conveys electrolytes with their plasma concentrations to the electrical double layer. But the concentrations in the effluent on the other side of that layer are changed by the Donnan effect to the equilibrium concentrations, independent of the filtration rate. When the extracted plasma volume is substituted by (dialysate) fluid with the equilibrium concentrations, the electrolyte balance is not disturbed. This is the answer to concern 1 formulated in Section 5.1.

#### 5.2.2. The Gibbs-Donnan Effect and Rising Protein-Concentration in Post-HDF

In treatments with high-volume-HDF in post-dilution, a large part of the plasma water is extracted in crossflow filtration while the blood is running through the capillaries. The electrolyte concentrations in the effluent filtrate have adapted to those of the Donnan equilibrium, which differ from the plasma concentrations by the Donnan factor *F_D_*. The local plasma electrolyte concentrations change steadily along the capillary flow path *x* with the increasing plasma filtration fraction *FF_p_(x)*. Cations are partially retained, so their plasma concentrations rise. The non-permeable proteins are retained in the residual plasma water, so their plasma concentrations rise, too. Anions are partially expelled from the plasma by the electrical forces in the double-layer, so their plasma concentrations diminish along the capillary flow path *x*. These independent concentration changes alter the local Donnan factor of the plasma (see Equation (23)) along the capillaries as the filtration fraction *FF_p_(x)* increases.

This was the reason for the concern that the electrolyte concentration of the Donnan equilibrium *C_i.Donnan_* could change accordingly. When the dialysate concentration equals the equilibrium concentration at the inlet of the dialyzer, they would differ downstream when the equilibrium concentration changes. This would require an intricate adaptation of the prescription of the dialysate electrolyte composition in order to achieve a therapeutic goal, e.g., to have a zero diffusive exchange of sodium.

The problem was analyzed in a theoretical model. The target filtration fraction *FF_p_* was sliced into small, finite fractions *∆FF_p_* to investigate whether, under the changing conditions along the capillaries, the equilibrium concentrations *C_i.Donnan_* of the electrolytes are affected. In every filtration fraction slice *∆FF_p_*, starting from the blood inlet to the outlet of the dialyzer, the following steps were performed:

(1) The filtration fraction *FF_p_(x)* of the plasma is incremented by a finite amount *∆FF_p_*.
(2) Calculation of the progressive thickening of bulk plasma proteins *C_P_* in the slice.
(3) Calculation of the changed plasma concentration of the electrolytes *C_i.p_*: Anions are depleted, and cations are concentrated by filtration due to the concentration differences between plasma and filtrate, having Donnan equilibrium concentrations.
(4) Calculation of the new, decreased Donnan factor *F_D_*.
(5) Calculation of the Donnan-equilibrium concentrations of the electrolytes *C_i.Donnan_* on the dialysate site.
(6) Go to step (1) until the target filtration fraction *FF_p_* is reached.

The intriguing result was that the equilibrium concentrations *C_i.Donnan_* are not noticeably changed by the filtration rate *Q_f_* and the consequently altered electrolyte compositions of the plasma. The impact remains vanishingly low even at extremely high plasma filtration fractions *FF_p_* up to 0.7, which are not reached even in high-volume HDF. Model-based analysis suggests that routine adjustment of dialysate electrolyte composition solely based on convective volume may not be necessary under standardized conditions; however, individualized clinical prescription remains essential. Regarding electrolyte prescription (Na, K, and HCO_3_^−^), including divalent ions such as calcium and magnesium, final adjustment remains the responsibility of the prescribing physician and should be individualized according to the patient’s clinical status, biochemical profile, and treatment response. In routine practice, typical dialysate concentrations are approximately 136–138 mmol/L for sodium, 2–3 mmol/L for potassium, 30–32 mmol/L for bicarbonate, 1.25–1.50 mmol/L for calcium, and 0.5–0.75 mmol/L for magnesium. However, specific adjustments, particularly for divalent ions, may be required depending on the composition of the acidifying buffer used in the dialysate (e.g., citric acid). Citrate can chelate calcium and magnesium, thereby reducing their bioavailable fraction and influencing transmembrane mass transfer. This interaction should be considered when prescribing dialysate composition, especially in patients at risk of electrolyte imbalance or altered mineral metabolism. In addition, the addition of glucose in the dialysate (typically around 5.5 mmol/L) should be considered to prevent glucose losses during treatment and to limit additional metabolic and nutritional stress.

**Figure 17:**
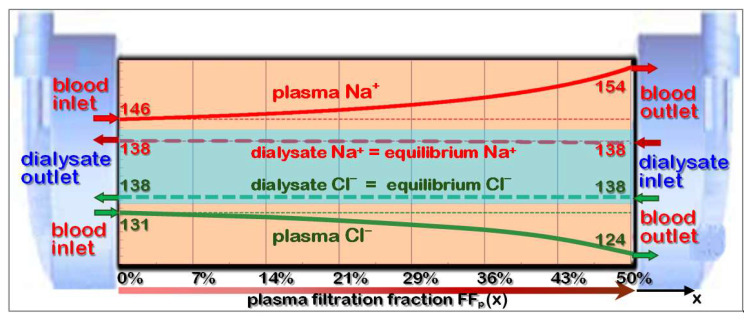
Concentration change in plasma electrolytes along the capillaries caused by the Donnan effect and by filtration (range of plasma filtration fraction *FF_p_*: 0% … 50% along the dialyzer). Red lines: Changes in plasma (full line) and equilibrium (dashed line) concentrations of sodium (cation, *z* = +1). Green lines: Changes in plasma (full line) and equilibrium (dashed line) concentrations of chloride (anion, z = −1)); Donnan factor decreases from blood inlet (*FF_p_* = 0%) to outlet (*FF_p_* = 50%) from 0.945 to 0.896, and the protein concentration doubles as 50% of plasma volume (brown arrow) is extracted. Red arrows indicate the flow direction of sodium. Green arrows indicate the flow direction of chloride.

For further illustration, the following example is considered with a simple plasma water and dialysate composition containing only the electrolytes Na^+^ and Cl^−^. The concentrations at the dialysate inlet are set equal to the Donnan equilibrium concentration of the blood inlet composition. No diffusive solute exchange happens with this condition. The selected input and the resulting output concentrations of the electrolytes are shown in Table 3, and Figure 17 depicts the concentrations in the dialyzer capillaries and in dialysate.

In high-volume HDF, the total plasma fraction removal is typically *FF_p_* = 0.5 as selected in this example. The sodium concentration of plasma water increases from 146 mmol/L to 154 mmol/L while the protein concentration doubles. On the other hand, the plasma water chloride concentration decreases from 131 mmol/L to 124 mmol/L (see Table 3 and Figure 17).

At the blood inlet of the dialyzer, the electrolytes are in a Donnan equilibrium with a factor of *F_D_* = 0.945. Despite the big concentration changes on the blood side, the equilibrium concentrations of 138 mmol/L remain constant for both electrolytes up to the dialyzer blood outlet, where the Donnan factor reaches its lowest value of *F_D_* = 0.896. This is shown by the following calculations: *C_i_*_,*Donnan*_ *= C_i_*_,*p*_ * *F_D_^zi^*.

The sodium and chloride concentrations in the dialysate are not changed from the selected inlet value of 138 mmol/L along the dialyzer, because they match the Donnan equilibrium concentrations. There is no diffusive electrolyte transfer across the membrane.

The protein and electrolyte concentrations of the blood plasma at the outlet of the dialyzer differ much from the original plasma composition of the patient, as shown in Figure 17 and in the data of Table 4. However, the concentrated plasma is diluted by infusing substitution fluid downstream of the dialyzer, restoring the electrolyte plasma concentrations to the patient’s levels before it is returned.

With a plasma filtration fraction of *FF_p_* = 50% as in the example above, the volumes of plasma and substitute are mixed in a 1:1 ratio. Dialysate and online-prepared substitutes have identical electrolyte compositions. Therefore, the electrolyte concentrations in the plasma after mixing yield the mean concentrations:

(25) $$C_{i,p,mix}=\left(C_{i,p,out}+C_{i,d}\right)/2$$

with

C_i,p,mix_: Electrolyte plasma concentrations after mixing;
C_i,p,out_: Electrolyte plasma concentrations at the blood outlet;
C_i,d_: Electrolyte concentrations in dialysate and substituate.

The concentrations in plasma after mixing (dilution) with substituate can be calculated using Equation (25):

Sodium: *C_Na_*_,*p*,*mix*_ = (154 + 138)/2 = 146 mmol/L = *C_Na_*_,*p*,0_;
Chloride: *C_Cl_*_,*p*,*mix*_ = (124 + 138)/2 = 131 mmol/L = *C_Cl_*_,*p*,0_;
Protein: *|z_p_|* * *C_P_*_,*mix*_ = (30 + 0)/2 = 15 mmol/L = *C_p_*_,*p*,0_.

In summary, the plasma water electrolyte concentrations returned to the patient in the venous line are identical to those in the arterial blood. The substitution fluid not only dilutes the concentrated proteins to the patient’s levels but resets the plasma electrolyte concentrations back as well.

## 6. Advancing Membrane Innovation: Future Directions and Emerging Trends

Even under optimistic assumptions, membranes that fully mimic the native kidney’s exchange capabilities are unlikely to emerge in the foreseeable future. The active transport of proteins or other solutes in an artificial membrane remains unfeasible. However, an innovative approach has been developed in the bioartificial kidney (BAK), where enough active immortalized tubular cells are incorporated into the dialysate compartment, restoring endocrine and metabolic functions. BAK has shown promising results in acute kidney injury patients requiring kidney support, with notable improvements in outcomes, yet its complexity and cost make it impractical for chronic kidney replacement due to scalability challenges.

**Figure 18:**
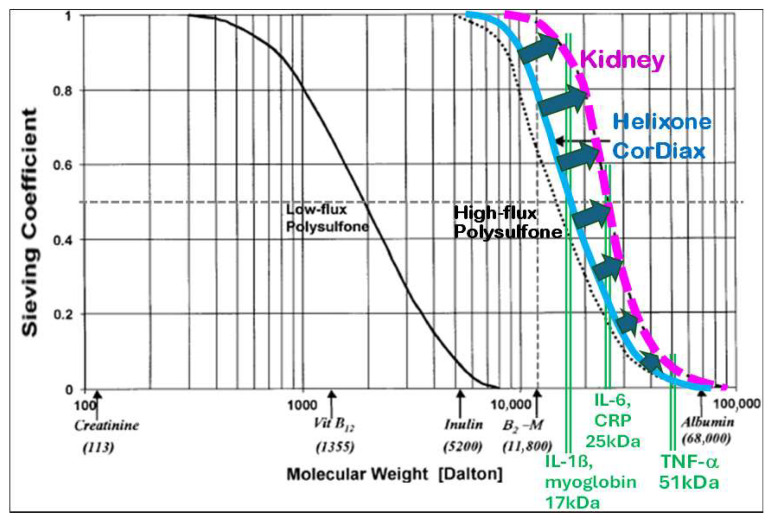
Enhancing the removal of large middle molecules by biomimicry of the kidneys.

A more realistic improvement lies in biomimicry, aiming to match the sieving characteristics of artificial membranes to those of native kidneys. To achieve this, retention must be shifted to higher molecular weights, without altering the albumin sieving coefficient, which already mimics that of the native kidneys. This requires the sieving coefficient curve, as shown in Figure 18, to be steeper and not just shifted. Another appealing approach to further enhance the efficiency of extracorporeal kidney replacement therapy is to develop multilayer membranes with adsorption capacity, focusing on protein-bound uremic toxins. Further progress in the field requires continuous efforts to improve the controlled quality of raw materials, such as artificial polymers and solvents, and refine manufacturing processes, including membrane and filter production and sterilization techniques, within economic constraints. While significant strides have been made, only incremental improvements are likely in the near future.

## 7. Conclusions

Advanced dialysis membranes, incorporating novel polymers and nanotechnology-based fiber-spinning techniques, combined with advanced HDF monitoring technologies, significantly enhance the efficacy of hemodiafiltration by enabling more efficient and selective removal of uremic toxins. Additionally, the hemocompatibility of membranes may be enhanced by modifying the surface-blood interface. Surface modification technologies can create a top layer that reduces the activation of the complement and coagulation systems, potentially eliminating or significantly lowering the need for heparin dosing.

Looking ahead, current technologies are sufficiently mature to enable the production of multilayer hollow fibers, with each layer designed to serve distinct functions. The innermost layer is critical for hemocompatibility, while its pore size distribution determines the molecular cut-off region for middle molecules and the albumin sieving coefficient. Subsequent layers can incorporate adsorptive materials designed to capture a broad range of organic uremic solutes, particularly protein-bound uremic toxins. As solutes diffuse through the inner layer, they can be captured within these outer adsorptive layers, offering high clearance efficiency even at relatively low dialysate flow rates. This approach combines diffusive transport with adsorption, for example, through the use of activated carbon to bind uremic solutes, including protein-bound compounds. Such adsorption can be implemented either via additional sorbent cartridges or, preferably, integrated directly into multilayer membrane architectures incorporating activated carbon.

Such technology could be integrated into a dialysate regeneration system and may even be used in the development of portable artificial kidneys. Furthermore, innovative approaches could enable the miniaturization of hydraulic systems to achieve compact, portable dialysis units. However, it is important to recognize that improving efficiency is not solely reliant on enhancing membrane permeability and solute clearance. Increasing treatment time and frequency also holds the potential for immediate positive outcomes, and this approach is already feasible.

## Figures and Tables

**Table 1:** Comparison of UFC (**a**) and sieving coefficients (**b**) of 2 dialyzer series.

**(a)**		
**Type (Area)**	**Ultrafiltration Coefficient [mL/h/mmHg]**	
	**ELISIO-H**	**SOLACEA-H**
**15H** (1.5 m^2^)	67	61
**17H** (1.7 m^2^)	74	69
**19H** (1.9 m^2^)	76	72
**21H** (2.1 m^2^)	82	76
**25H** (2.5 m^2^)	93	87
**(b)**		
**Molecule (MW [kDa])**	**Sieving Coefficient [1]**	
	**ELISIO-H**	**SOLACEA-H**
**Vitamin B12** (1.4)	0.989	1.00
**Inulin** (5.2)	0.926	1.00
**ß2m** (11.8)	0.803	0.85
**Myoglobin** (17.0)	0.223	0.80
**Albumin** (67.0)	0.002	0.013

**Table 2:** Reduction in middle molecules and albumin loss in Expanded HD [57,58].

Reduction Rate of Middle Molecules and Albumin Loss	MW [kDa]	Theranova 400^TM^ 1.8 m^2^	Theranova 500^TM^ 2.0 m^2^
ß2-microglobulin	12	78%	74.7%
Myoglobin	17	68%	62.5%
k–free light chains/Prolactin	23	70%	60.0%
YKL-40/a1-glycoprotein	40	63%	2.8%
loss of albumin	67	3.0 ± 0.2 g/Tx	0.03 ± 0.01 g/Tx

**Table 3:** Inlet composition of plasma and dialysate electrolytes used for simulation and resulting outlet concentration at a filtration fraction of *FF_p_* = 0.50. (p = plasma, d = dialysate).

Ion i	Na,p	Cl,p	Protein	F_D_	Na,d	Cl,d
**z_i_**	+1	−1	−15	- -	+1	−1
**C_i,inlet_** [mmol/L]	146	131	1.0	0.945	138	138
**C_i,outlet_** [mmol/L] @ FF_p_ = 0.50	154	124	2.0	0.896	138	138

**Table 4:** Calculation of equilibrium concentrations of the Donnan effect at plasma compositions and Donnan factors at the inlet and the outlet (*FF_p_* = 0.50) of the dialyzer.

Electrolyte	Blood Inlet	Blood Outlet (FF_p_ = 0.50)	Equilibrium Concentration
Sodium (Na^+^)	146 mmol/L * 0.945 =	154 mmol/L * 0.896 =	138 mmol/L
Chloride (Cl^−^)	131 mmol/L/0.945 =	124 mmol/L/0.896 =	138 mmol/L

## Data Availability

The original contributions presented in this study are included in the article material. Further inquiries can be directed to the corresponding author.
